# CyberShield analytics: SHAP-guided stacking ensembles for organizational risk resilience

**DOI:** 10.3389/frai.2026.1878587

**Published:** 2026-10-09

**Authors:** Sadiya Chowdhury, Abdullah Al Imtihan

**Affiliations:** 1 Water Supply and Sanitation Authority, Dhaka, Bangladesh; 2 European Institute for Materials AI & Technology, Madrid, Spain

**Keywords:** cyber resilience, explainable AI, feature engineering, network intrusion detection, SHAP, stacking ensemble, statistical validation, UNSW-NB15

## Abstract

**Introduction:**

The escalating sophistication of cyber threats against organizational infrastructure demands detection frameworks that simultaneously achieve high classification performance, statistical robustness, and operational transparency. Most published intrusion detection frameworks optimize for accuracy alone, omitting leakage-free evaluation protocols, formal significance testing, and the auditable explanations required by contemporary AI governance frameworks.

**Methods:**

This paper presents CyberShield Analytics, a SHAP-guided two-level stacking ensemble framework for network intrusion detection and organizational risk resilience. The study addresses the binary intrusion-detection task (Attack vs. Normal); the 10 underlying traffic categories of the dataset are used only for exploratory characterization and are not predicted as separate classes. Evaluated on the benchmark UNSW-NB15 dataset comprising 257,673 network flow records, the framework integrates nine heterogeneous base learners-Logistic Regression, Decision Tree, Random Forest, Extra Trees, Gradient Boosting, XGBoost, LightGBM, Multilayer Perceptron, and K-Nearest Neighbors-with an XGBoost meta-learner operating on leakage-free out-of-fold probability estimates. A systematic feature engineering pipeline expands the original 45-feature space to 67 dimensions through 22 domain-informed interaction and transformation features.

**Results:**

The ensemble achieves Accuracy = 0.9535, *F*_1_ = 0.9535, and AUC-ROC = 0.9929 on a fully held-out test set of 51,535 instances, outperforming all individual base learners. Statistical validity is confirmed via 5-Fold Stratified Cross-Validation (mean *F*_1_ = 0.9499 ± 0.0013), with paired *t*-tests showing significant superiority over XGBoost (*t* = 7.165, *p* = 0.002), LightGBM (*t* = 25.108, *p* < 0.0001), and Extra Trees (*t* = 3.178, *p* = 0.034). Error evaluation confirms the lowest MAE (0.04653) and RMSE (0.21571) among all evaluated models. A tri-method Explainable AI (XAI) layer comprising SHAP, DeepLIFT, and Integrated Gradients applied to representative base learners consistently identifies sttl, ct_dst_sport_ltm, and byte_ratio as the dominant threat-discriminating features.

**Discussion:**

The CyberShield framework bridges the critical gap between predictive performance and operational explainability. Improvements should be read as gains in robustness, calibration, and statistical reliability at comparable accuracy rather than a headline accuracy increase. The framework offers actionable threat intelligence for Security Operations Centers (SOCs) and cyber risk governance in enterprise environments.

## 1 Introduction

The global cyber threat landscape has undergone a paradigm shift in scope and severity over the past decade. According to the IBM Cost of a Data Breach Report, the average cost of a data breach reached $4.88 million in 2024, with network intrusions comprising the majority of incident vectors (IBM Security, 2024). Organizations across financial services, critical infrastructure, and healthcare sectors face increasingly adaptive adversaries deploying multi-vector attacks including Denial-of-Service (DoS), Exploits, Reconnaissance, and Advanced Persistent Threats (APTs) (Sarker, 2024). Traditional perimeter-based security mechanisms—firewalls, signature detection, and rule-based Intrusion Detection Systems (IDS)—are fundamentally ill-suited to detect zero-day exploits and polymorphic malware, as they rely on static pattern libraries that cannot adapt to novel attack vectors (Sharma and Gupta, 2024).

Machine learning (ML) has emerged as a transformative paradigm for anomaly-based network intrusion detection, enabling models to learn discriminative representations from high-dimensional network flow data (Chen and Li, 2025; Al-Haija and Droos, 2024). Supervised ensemble methods—particularly Random Forest, XGBoost, and LightGBM—have demonstrated strong classification performance on benchmark datasets, yet each individual learner exhibits characteristic failure modes: tree-based methods suffer from high variance; gradient boosting is computationally intensive; and neural networks remain opaque black boxes offering no interpretable decision rationale for security analysts (Capuano et al., 2022; Demšar, 2006). *Stacking generalization*—a meta-learning strategy wherein a higher-level learner is trained on the out-of-fold predictions of diverse base models—has been shown to mitigate individual learner weaknesses while preserving their collective discriminative strength (Wolpert, 1992; Rajagopal et al., 2020).

Despite these advances, two critical gaps persist in the existing literature. First, the majority of published IDS frameworks optimize solely for classification accuracy, neglecting the organizational requirement for *transparent and auditable* AI decisions. Regulatory frameworks such as the EU AI Act, NIST AI RMF, and ISO/IEC 42001:2023 explicitly mandate explainability for high-risk AI deployments in security-critical environments (Barredo-Arrieta et al., 2020; Panigutti et al., 2023). Second, reported performance improvements are rarely subjected to rigorous statistical significance testing, rendering claimed superiority claims potentially attributable to random variation across experimental folds rather than genuine model improvements (Demšar, 2006). These limitations collectively undermine the operational deployment readiness and governance compliance of published IDS models.

This paper addresses both gaps through the following contributions:

1. **CyberShield stacking ensemble:** A two-level stacking architecture with nine heterogeneous base learners and an XGBoost meta-learner, achieving AUC-ROC = 0.9929 and *F*_1_ = 0.9535 on the UNSW-NB15 benchmark dataset.
2. **Domain-informed feature engineering:** A 22-feature expansion from 45 to 67 dimensions using interaction terms, logarithmic transforms, and ratio features, all constructed without data leakage by applying transformations exclusively to training-partition statistics.
3. **Leakage-free evaluation protocol:** A rigorous pipeline-encapsulated evaluation framework in which all preprocessing, scaling, and meta-feature generation operations are strictly contained within CV fold boundaries, preventing distributional leakage.
4. **Tri-method XAI layer:** A unified explainability framework integrating SHAP (Lundberg and Lee, 2017), DeepLIFT (Shrikumar et al., 2017), and Integrated Gradients (Sundararajan et al., 2017), providing cross-method attribution stability validation and actionable feature rankings for SOC deployment.
5. **Rigorous statistical validation:** Performance superiority is verified via Wilcoxon Signed-Rank Test and Paired *t*-Test across five cross-validation folds, confirming significance at α = 0.05 against three of four baselines.

### 1.1 Novelty and positioning

Unlike prior stacking-based IDS studies, which typically combine three to four homogeneous tree learners and report only point-estimate accuracy on a single split, the distinguishing contribution of CyberShield is the *simultaneous* integration of (i) a nine-learner heterogeneous base layer spanning linear, tree-based, gradient-boosted, neural, and instance-based model families; (ii) a strictly pipeline-encapsulated, leakage-free evaluation protocol applied uniformly to preprocessing, feature engineering, and out-of-fold meta-feature generation; (iii) a tri-method XAI layer whose cross-method consensus is treated as evidence of genuine feature reliance; and (iv) formal statistical hypothesis testing of the performance gain. To our knowledge, no prior UNSW-NB15 stacking study combines all four elements; existing works (Table 1) satisfy at most two. The contribution is therefore methodological rigor and operational auditability rather than a raw accuracy record.

**Table 1:** Positioning of CyberShield analytics relative to prior work.

Study	Ensemble	XAI	Stat. test	No leakage	Multi-XAI
Kasongo and Sun (2020)	✓	×	×	Partial	×
Rajagopal et al. (2020)	✓	×	×	Partial	×
Senevirathna et al. (2024)	✓	×	×	Partial	×
Vishwakarma et al. (2025)	✓	SHAP	×	Partial	×
Warnecke et al. (2020)	×	SHAP	×	✓	Partial
**CyberShield (Ours)**	✓	✓	✓	✓	✓

Multi-XAI: SHAP + DeepLIFT + Integrated Gradients combined.

The remainder of this paper is structured as follows. Section 2 reviews related work in ensemble-based IDS and XAI-augmented cybersecurity. Section 3 details the proposed methodology. Section 4 presents and analyses experimental results. Section 6 concludes the paper and outlines future research directions.

## 2 Literature review

### 2.1 Traditional intrusion detection systems

Early Network Intrusion Detection Systems (NIDS) relied on signature-based approaches, matching observed network traffic against pre-compiled attack libraries (Sharma and Gupta, 2024). While effective against known attack patterns, these systems fundamentally cannot detect zero-day exploits or novel variants of existing attack families. Anomaly-based IDS emerged to address this limitation by establishing statistical baselines of normal behavior and flagging statistically significant deviations (Liu et al., 2012; Goldstein and Uchida, 2016). Early anomaly detectors employed statistical methods—Gaussian mixture models, principal component analysis, and support vector machines—but their reliance on hand-crafted feature engineering and linear separability assumptions limited their effectiveness on high-dimensional, non-linear network traffic data (Chen and Li, 2025).

### 2.2 Machine learning approaches for IDS

The proliferation of benchmark datasets such as KDD Cup 1999, NSL-KDD, and UNSW-NB15 (Moustafa and Slay, 2015) accelerated the adoption of supervised ML for IDS. Kasongo and Sun (Kasongo and Sun, 2020) demonstrated that Random Forest with wrapper-based feature selection achieved 93.62% accuracy on UNSW-NB15. Deep learning approaches, particularly Convolutional Neural Networks (CNNs) and Long Short-Term Memory (LSTM) networks, have demonstrated superior feature abstraction capabilities. However, these deep architectures require significant training data, computational resources, and—critically—sacrifice interpretability for performance gains (Capuano et al., 2022; Marino et al., 2018; Lee et al., 2022).

Multiclass classification on UNSW-NB15 has been studied by several groups. Rajagopal et al. (2020) demonstrated that a stacking ensemble applied to heterogeneous datasets (UNSW-NB15 and UGR'16) achieved 94%–97% accuracy, outperforming individual classifiers. More recently, Vishwakarma et al. (2025) reported that an explainable stacking ensemble improved model transparency without sacrificing classification accuracy.

### 2.3 Ensemble methods and stacking generalization

Stacking generalization, introduced by Wolpert (1992) and subsequently formalized by Breiman (1996), addresses the bias-variance tradeoff by training a meta-learner on the out-of-fold predictions of diverse base classifiers. The present work systematically extends these contributions by incorporating nine diverse base learners, enforcing strict anti-leakage pipeline encapsulation within every cross-validation fold, and integrating a tri-method XAI layer for cross-validated explainability (Zhang et al., 2024).

### 2.4 Explainable AI in cybersecurity

The “black box” nature of high-performing ML models presents a fundamental operational challenge for security analysts who require not only accurate predictions but also justifiable, auditable decision rationales (Senevirathna et al., 2024; Barredo-Arrieta et al., 2020). SHAP (Lundberg and Lee, 2017), grounded in Shapley values from cooperative game theory, has emerged as the dominant post-hoc explanation method for tabular ML models in cybersecurity contexts (Lundberg et al., 2020; Rajpurkar et al., 2024). Warnecke et al. (2020); Pande et al. (2023) systematically evaluated SHAP, LIME, and gradient-based methods for deep learning IDS, finding that SHAP provided the most stable and semantically consistent explanations across attack categories.

DeepLIFT (Shrikumar et al., 2017) propagates activation differences relative to a reference baseline through the network in a single backward pass. Integrated Gradients (Sundararajan et al., 2017) provides axiomatic guarantees—Sensitivity and Completeness—by integrating gradients along a linear interpolation from baseline to input. Capuano et al. (2022) evaluated these methods for deep learning-based IDS and found that cross-method consensus provides stronger evidence of genuine model reliance than any single method alone—a validation principle directly adopted in the present work.

### 2.5 Organizational cyber risk resilience

Beyond detection accuracy, contemporary cyber risk governance frameworks. Jalalvand et al. (2024) survey criteria and methods for alert prioritization in security operations centres and report that interpretable risk metrics support more effective triage than opaque alternatives, directly informing the design philosophy of the CyberShield framework.

### 2.6 Research gaps and positioning

Table 1 summarizes the key limitations of prior work addressed by the present study.

## 3 Methodology

### 3.1 Dataset description

This study employs the **UNSW-NB15** benchmark dataset, generated by the Australian Center for Cyber Security (ACCS) using the IXIA PerfectStorm tool in a controlled Cyber Range Laboratory environment (Moustafa and Slay, 2015). The dataset comprises **257,673 network flow records** with **45 raw features** spanning nine attack categories—Fuzzers, Analysis, Backdoors, Denial-of-Service (DoS), Exploits, Generic, Reconnaissance, Shellcode, and Worms—alongside a Normal traffic class. Following stratified partitioning, 206,138 samples (80%) were allocated for training and 51,535 samples (20%) for testing. A preliminary data quality audit confirmed zero missing values, zero duplicate records, and a total memory footprint of 138.97 MB. The class composition of the dataset is shown in Figure 1, and the class-conditional distributions of the principal flow features are shown in Figures 2–5. Figure 6 reports the skewness and kurtosis of all 45 raw features, and Figure 7 shows the distributions of the engineered features by class Table 2.

**Figure 1:**
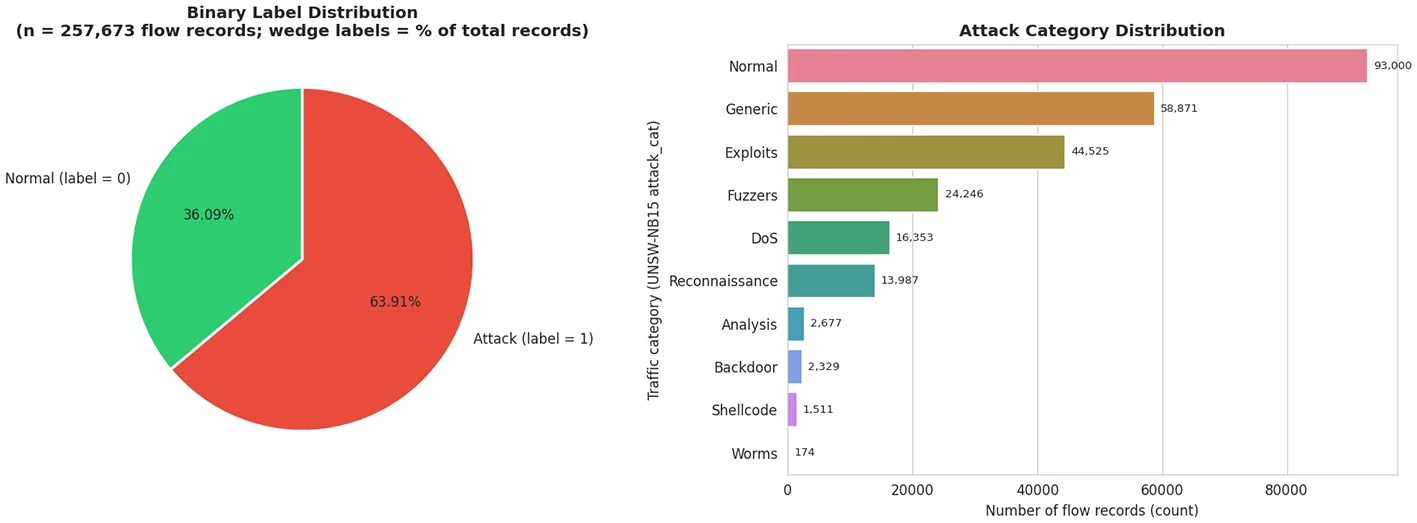
Class distribution of the UNSW-NB15 dataset. **Left**: Binary label distribution showing 63.91% malicious (Attack) and 36.09% benign (Normal) traffic. **Right**: Attack category distribution; Generic attacks constitute the largest class (58,871 samples, 35.7%).

**Figure 2:**
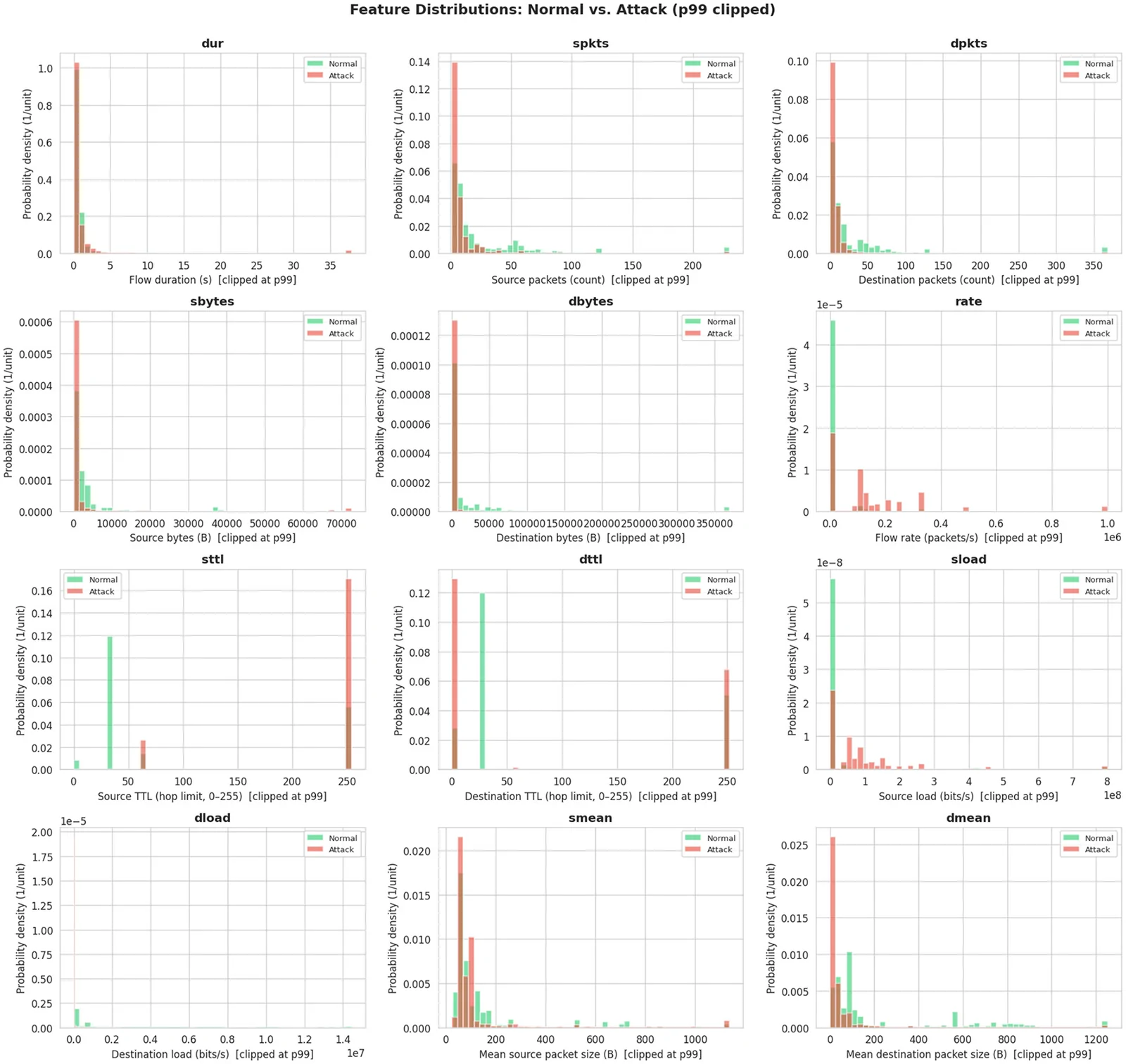
Feature distributions (p99-clipped) for key UNSW-NB15 features stratified by class (green = Normal, red = Attack).

**Figure 3:**
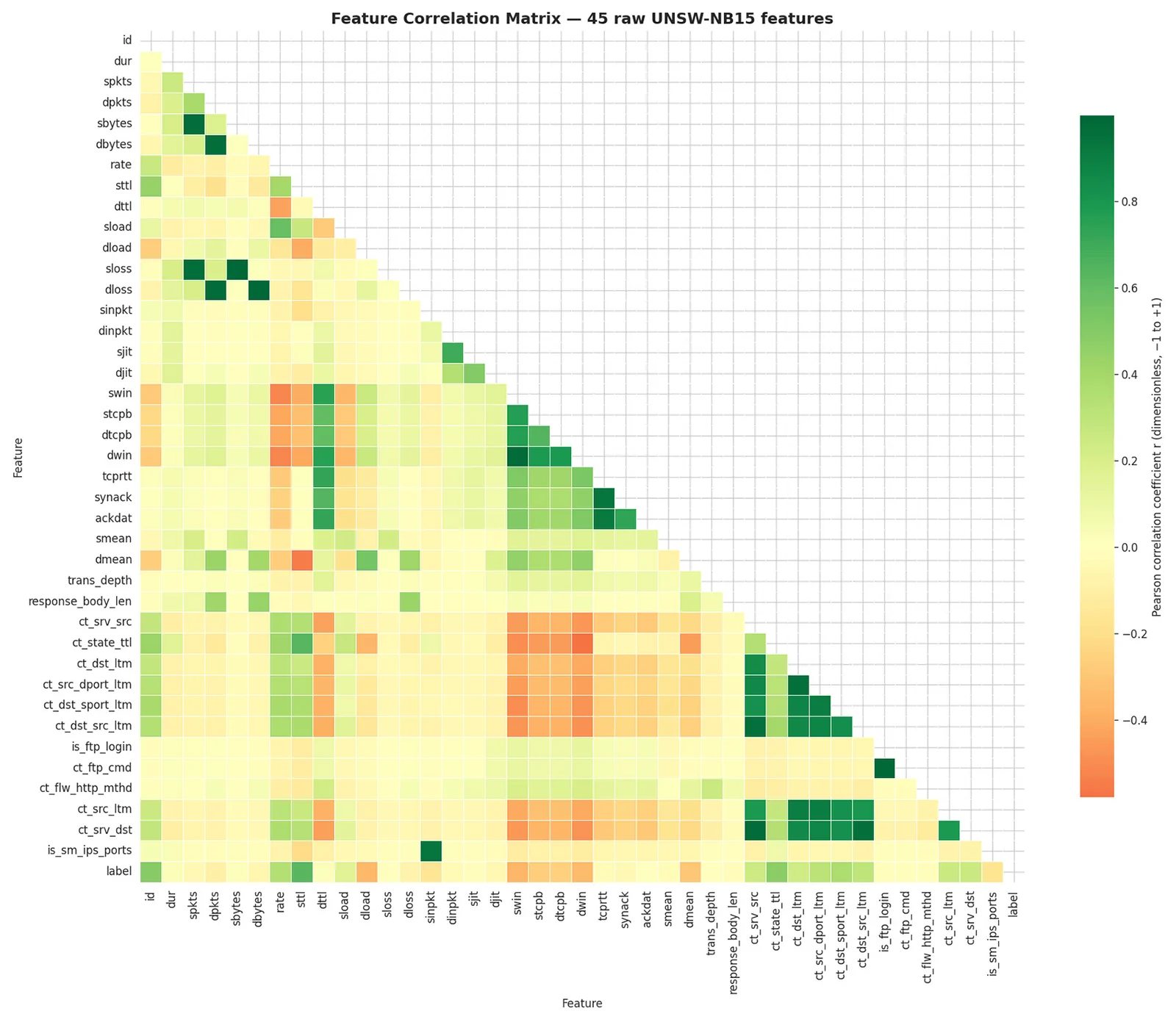
Pearson correlation matrix of the 45 raw UNSW-NB15 features.

**Figure 4:**
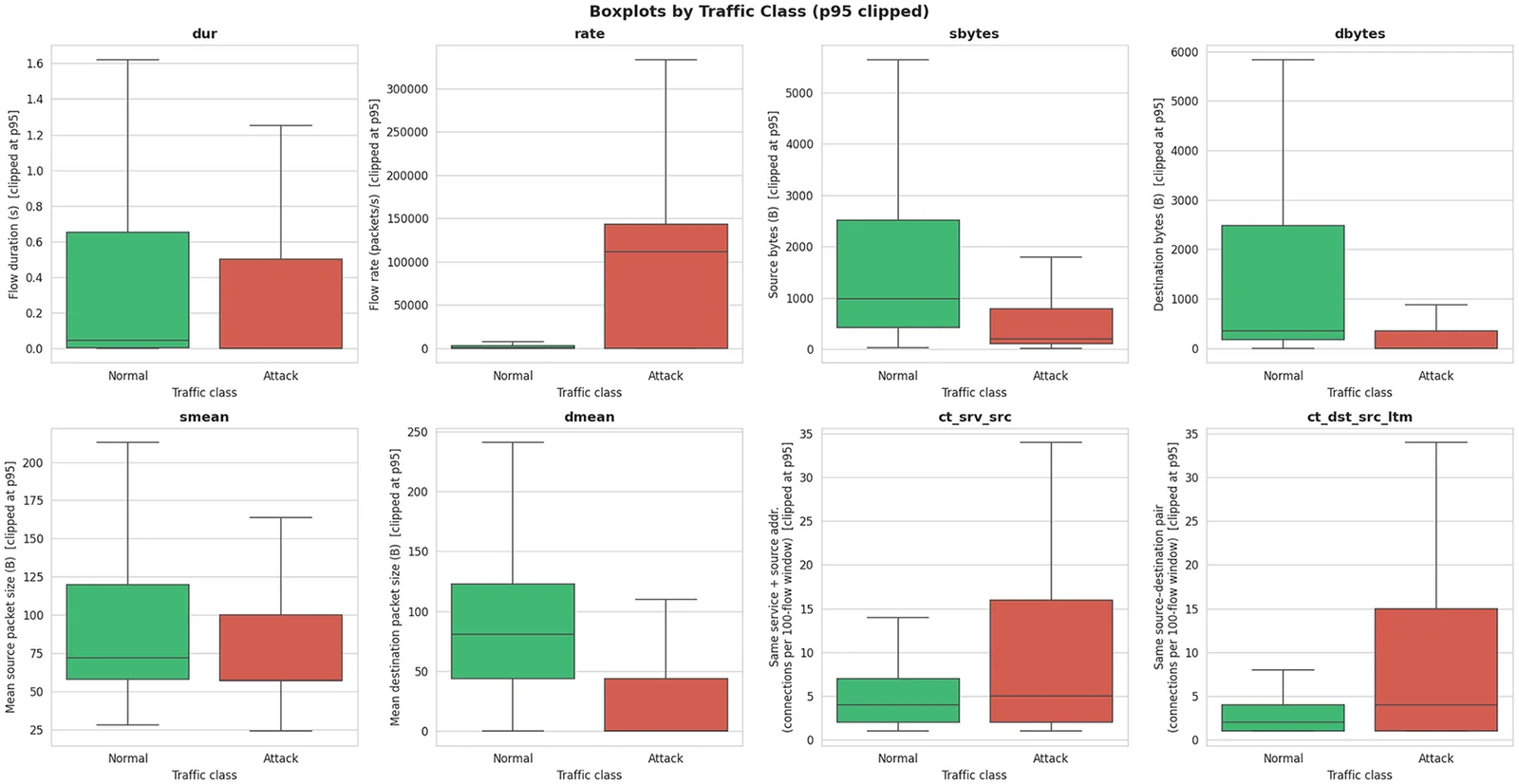
Boxplots (p95-clipped) for eight key UNSW-NB15 features stratified by class.

**Figure 5:**
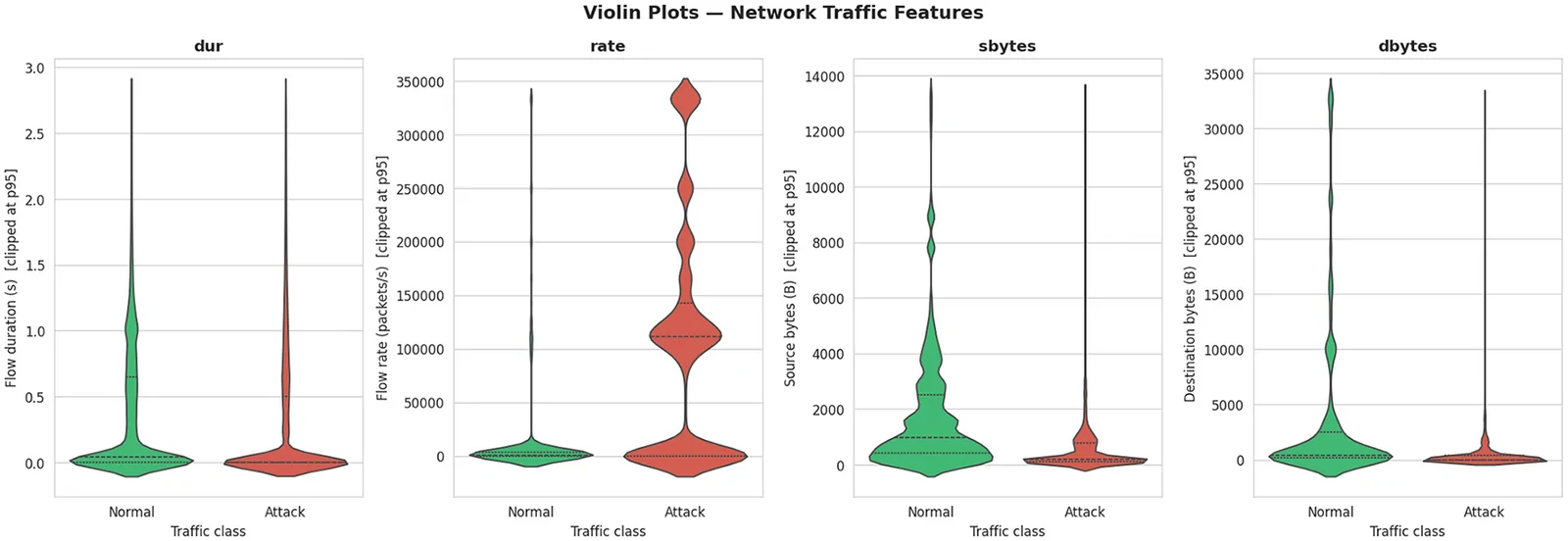
Violin plots for four key network flow features by class.

**Figure 6:**
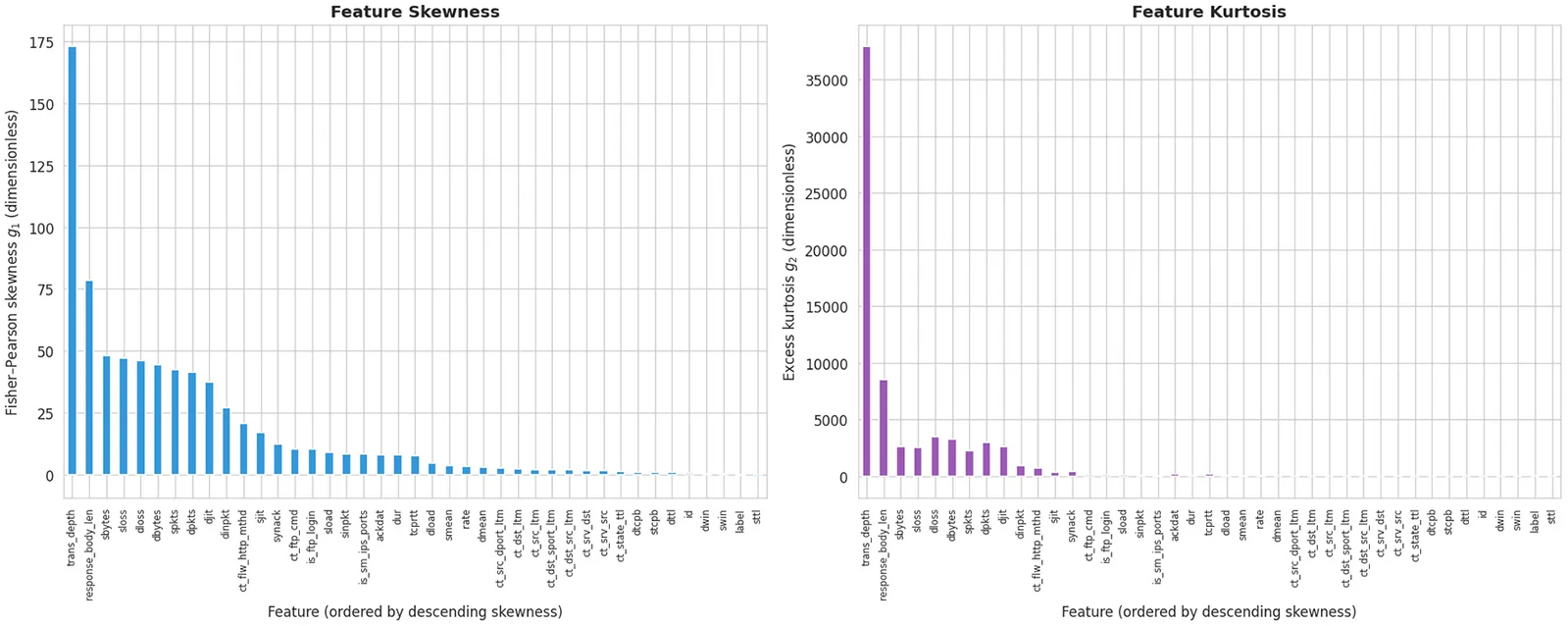
Feature skewness (**left**, blue) and kurtosis (**right**, purple) for all 45 raw features in descending order. trans_depth exhibits extreme skewness (173.16) and kurtosis (37,927.61).

**Figure 7:**
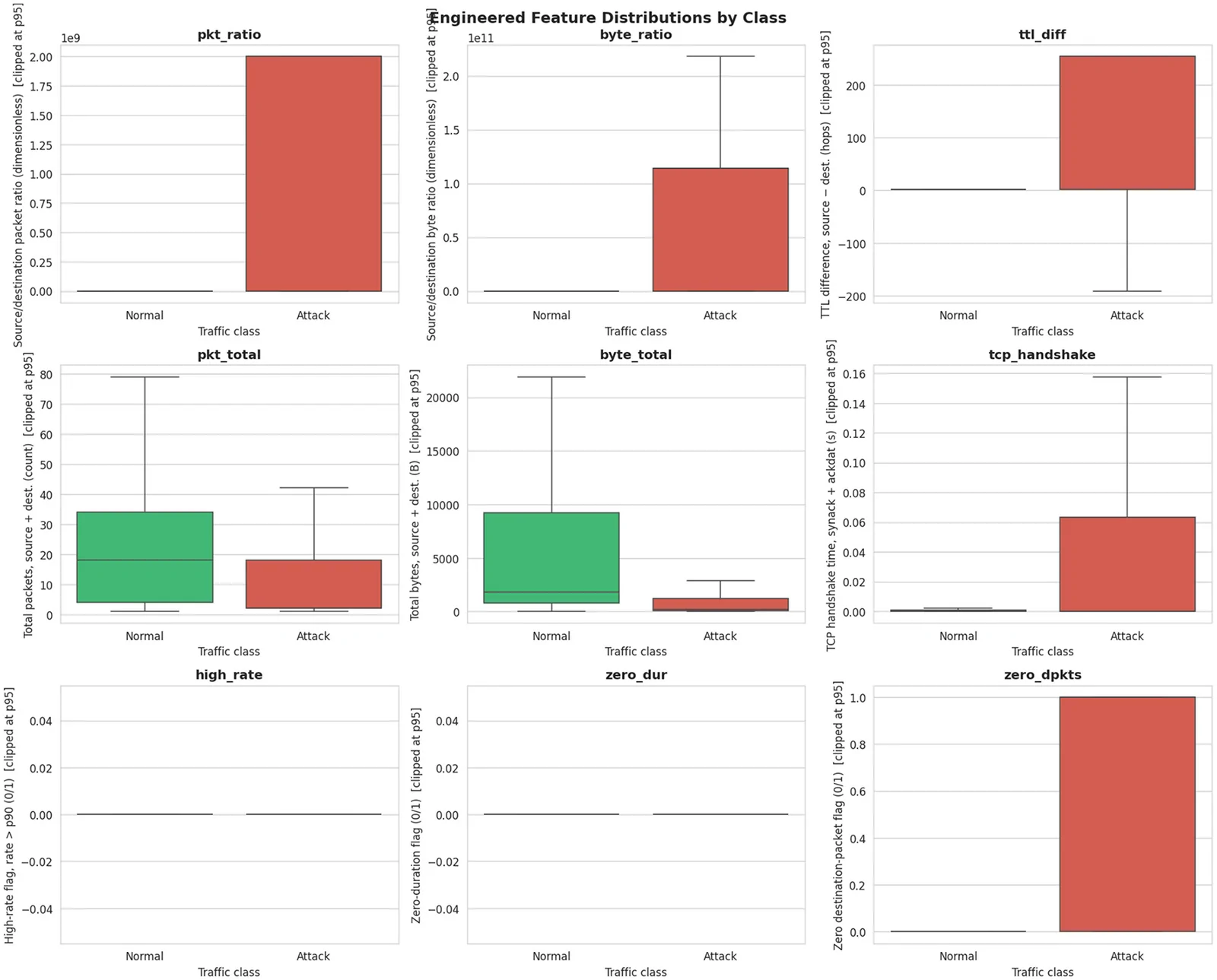
Boxplot distributions of nine engineered features by class.

**Table 2:** Statistical summary of selected UNSW-NB15 features.

Feature	Mean	Std. dev.	Min	Max	Skewness
dur	1.247	5.974	0	59.999	—
sbytes	8,572.95	173,773.88	24	14,355,774	47.92
dbytes	14,387.29	146,199.28	0	14,657,531	44.34
sttl	180.001	102.488	0	255	—
rate	91,253.91	160,344.64	0	1,000,000	—
trans_depth	0.102	0.711	0	172	173.16
response_body_len	1,968.90	49,625.23	0	6,558,056	78.48
tcprtt	0.046	0.093	0.000	3.821	—

#### 3.1.1 Classification task

Although UNSW-NB15 provides fine-grained multiclass labels (nine attack families plus Normal), the present study formulates and evaluates intrusion detection as a *binary* problem: each flow is classified as Attack (*y* = 1) or Normal (*y* = 0). The attack-category labels are retained only for the exploratory analysis of class prevalence in Section 4.1; all reported metrics, confusion matrices, ROC curves, and statistical tests pertain to the binary task. Accordingly, no claim is made regarding discrimination *among* individual attack categories. Multiclass attack-family classification is identified as future work in Section 6.

Several features exhibit severe positive skewness exceeding 40 (e.g., skew(sbytes) = 47.92, skew(sloss) = 47.12), necessitating logarithmic transformation during preprocessing.

### 3.2 Data preprocessing

Raw network flow data underwent a systematic six-step preprocessing pipeline designed to ensure representational integrity and eliminate data leakage (Pedregosa et al., 2011) (Equations 1, 2).

#### 3.2.1 Step 1—Identifier and timestamp removal

Non-informative identifiers (id) and timestamp-derived fields were excluded to prevent spurious temporal correlations.

#### 3.2.2 Step 2—Categorical encoding

Three categorical variables—proto (133 unique values), service (13), and state (11)—were encoded using Label Encoding after cardinality analysis.

#### 3.2.3 Step 3—Stratified train/test split

Data were partitioned *prior* to any feature engineering or scaling:


$$\begin{array}{l} D=D_{\text{train}}\cup D_{\text{test}},\;|D_{\text{train}}|=206,138,\;|D_{\text{test}}|=51,535. \end{array}$$ (1)

#### 3.2.4 Step 4—Logarithmic transformation

Features with absolute skewness exceeding 25 were log-transformed:


$$\begin{array}{l} x_{j}^{*}=\log\left(x_{j}+1\right),\;|\mathrm{Skew}\left(x_{j}\right)|>25. \end{array}$$ (2)

#### 3.2.5 Step 5—Standardization

A StandardScaler was fitted exclusively on $D_{\text{train}}$ (Equation 3):


$$\begin{array}{l} {\tilde{x}}_{ij}=\frac{x_{ij}-\mu_{j}^{\text{train}}}{\sigma_{j}^{\text{train}}}. \end{array}$$ (3)

#### 3.2.6 Step 6—Pipeline encapsulation

All preprocessing operations were encapsulated within an sklearn.Pipeline (Pedregosa et al., 2011).

#### 3.2.7 Class imbalance handling

The binary target is moderately imbalanced, with an Attack:Normal ratio of approximately 1.77:1 (63.91% Attack, 36.09% Normal). This is a mild imbalance that does not require aggressive rebalancing. Three complementary measures were nonetheless adopted. First, both the train/test split and the 5-fold cross-validation were *stratified*, preserving the marginal class distribution in every partition. Second, cost-sensitive learning was enabled where supported (class_weight = “balanced~ for Logistic Regression, Decision Tree, Random Forest and Extra Trees; scale_pos_weight for XGBoost/LightGBM), so that the minority Normal class contributes proportionally more to the loss. Third, threshold-independent and imbalance-robust metrics (*F*_1_ and AUC-ROC) were used as the primary selection criteria rather than raw accuracy. Synthetic minority over-sampling [SMOTE (Chawla et al., 2002)] was evaluated during development but, applied strictly inside the training folds, produced no statistically significant *F*_1_ gain while degrading probability calibration; it was therefore not retained in the final pipeline.

### 3.3 Feature engineering

To enrich the original 45-feature representation, 22 domain-informed engineered features were constructed exclusively from training-set statistics, expanding the feature space to 67 dimensions. The complete transformation pipeline is summarized in Figure 8 and Equations 4–11.

**Figure 8:**
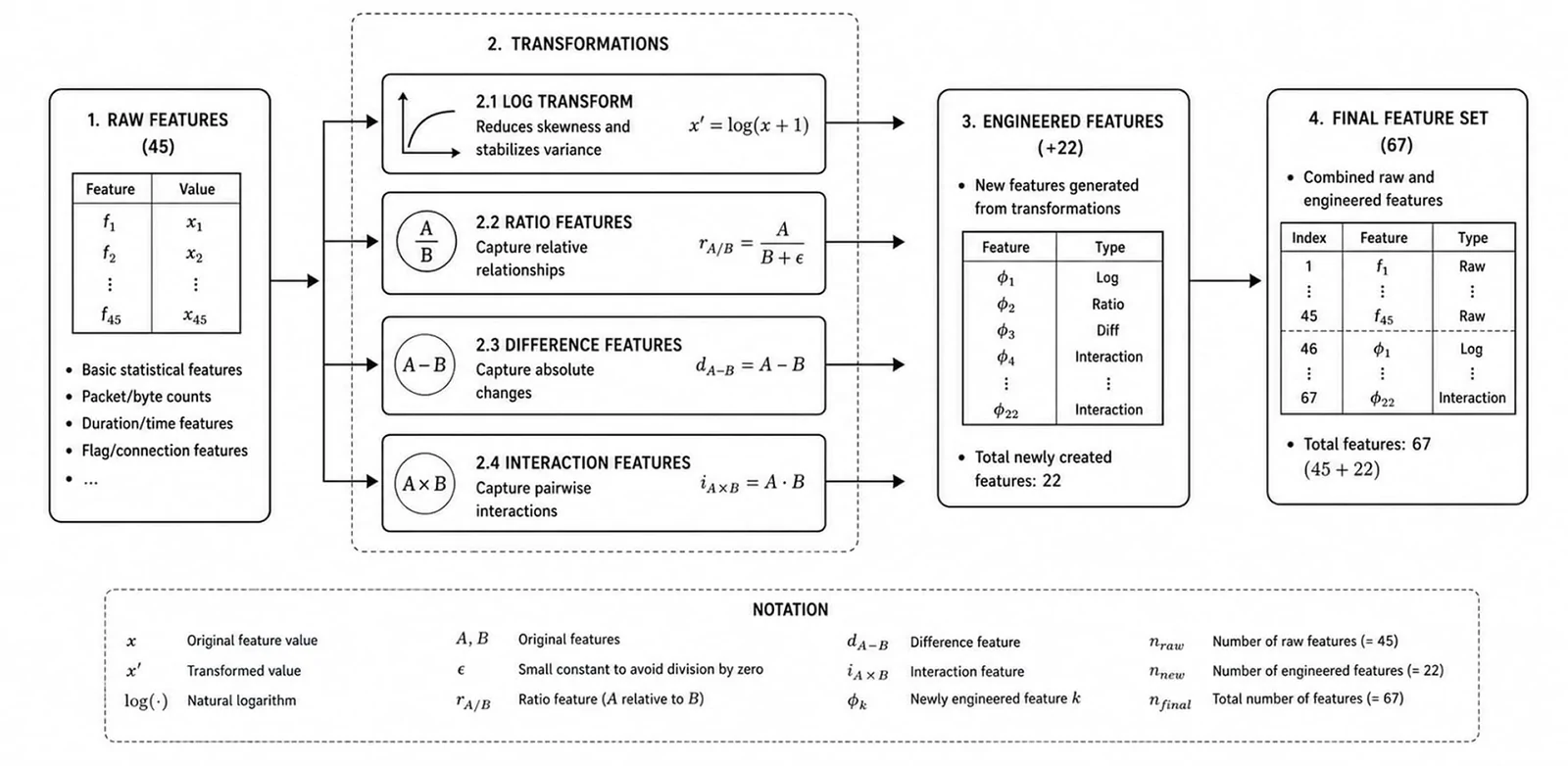
Feature engineering pipeline. Starting from 45 raw features, four transformation categories are applied exclusively to training-partition statistics: (2.1) log transforms *x*′ = log(*x* + 1); (2.2) ratio features *r*_*A*/*B*_ = *A*/(*B* + ϵ); (2.3) difference features *d*_*A*−*B*_ = *A* − *B*; (2.4) interaction features *i*_*A*×*B*_ = *A* · *B*.

#### 3.3.1 Rationale for the 22 features

The engineered set was not chosen arbitrarily but derived from four attack-motivated transformation families, each targeting a known limitation of the raw features for intrusion discrimination: (i) *logarithmic transforms* (*x*′ = log(*x* + 1)) applied to the heavily right-skewed volume features (skewness >25; see Table 3) to linearise their relationship with the decision boundary; (ii) *ratio features* (e.g., byte_ratio, Port_Ratio, Payload_per_Port) that capture source/destination asymmetry characteristic of exfiltration and scanning; (iii) *difference features* (e.g., mean_diff) encoding directional traffic imbalance; and (iv) *interaction terms* (e.g., Conf × Payload) combining connection-state and volume signals. Within each family, candidates were retained only if they (a) were motivated by an established attack signature and (b) exhibited non-redundant behavior, i.e. pairwise Pearson correlation |ρ| < 0.95 with existing features on the training partition. This procedure yielded 22 features. Their utility is confirmed *post hoc* in Section 4.6: three engineered features (byte_ratio, log_dbytes, mean_diff) rank within the top-10 global SHAP importances.

**Table 3:** Top-10 most skewed features in the UNSW-NB15 dataset.

Rank	Feature	Skewness	Kurtosis
1	trans_depth	173.16	37,927.61
2	response_body_len	78.48	8,513.82
3	sbytes	47.92	2,616.50
4	sloss	47.12	2,551.47
5	dloss	46.09	3,502.78
6	dbytes	44.34	3,272.35
7	spkts	42.52	2,213.27
8	dpkts	41.19	2,965.88
9	djit	37.50	2,638.49
10	dinpkt	26.97	939.57

Key interaction and transformation features are defined as follows:


$$\begin{array}{l} \text{Port\_Ratio}=\frac{x_{\text{sport}}}{x_{\text{dport}}+1}, \end{array}$$ (4)

$$\begin{array}{l} \text{Port\_Diff}=|x_{\text{sport}}-x_{\text{dport}}|, \end{array}$$ (5)

$$\begin{array}{l} \text{Payload\_per\_Port}=\frac{x_{\text{sbytes}}}{x_{\text{sport}}+1}, \end{array}$$ (6)

$$\begin{array}{l} \text{byte\_ratio}=\frac{x_{\text{sbytes}}}{x_{\text{dbytes}}+1}, \end{array}$$ (7)

$$\begin{array}{l} \text{log\_dbytes}=\log\left(x_{\text{dbytes}}+1\right), \end{array}$$ (8)

$$\begin{array}{l} \text{mean\_diff}=x_{\text{smean}}-x_{\text{dmean}}, \end{array}$$ (9)

$$\begin{array}{l} \text{Conf}\times \text{Payload}=x_{\text{conf}}\cdot x_{\text{sbytes}}, \end{array}$$ (10)

$$\begin{array}{l} \text{High\_Conf}=1\left[x_{\text{conf}}>0.75\right]. \end{array}$$ (11)

### 3.4 Stacking ensemble architecture

The proposed CyberShield framework employs a two-level stacking ensemble architecture (Wolpert, 1992; Breiman, 1996) (Equations 12–15).

Let $L_{0}$ denote the set of Level-0 base learners and $L_{1}$ the meta-learner. Figure 9 presents the complete end-to-end CyberShield Analytics pipeline, and Figure 10 details the two-level stacking architecture.

**Figure 9:**
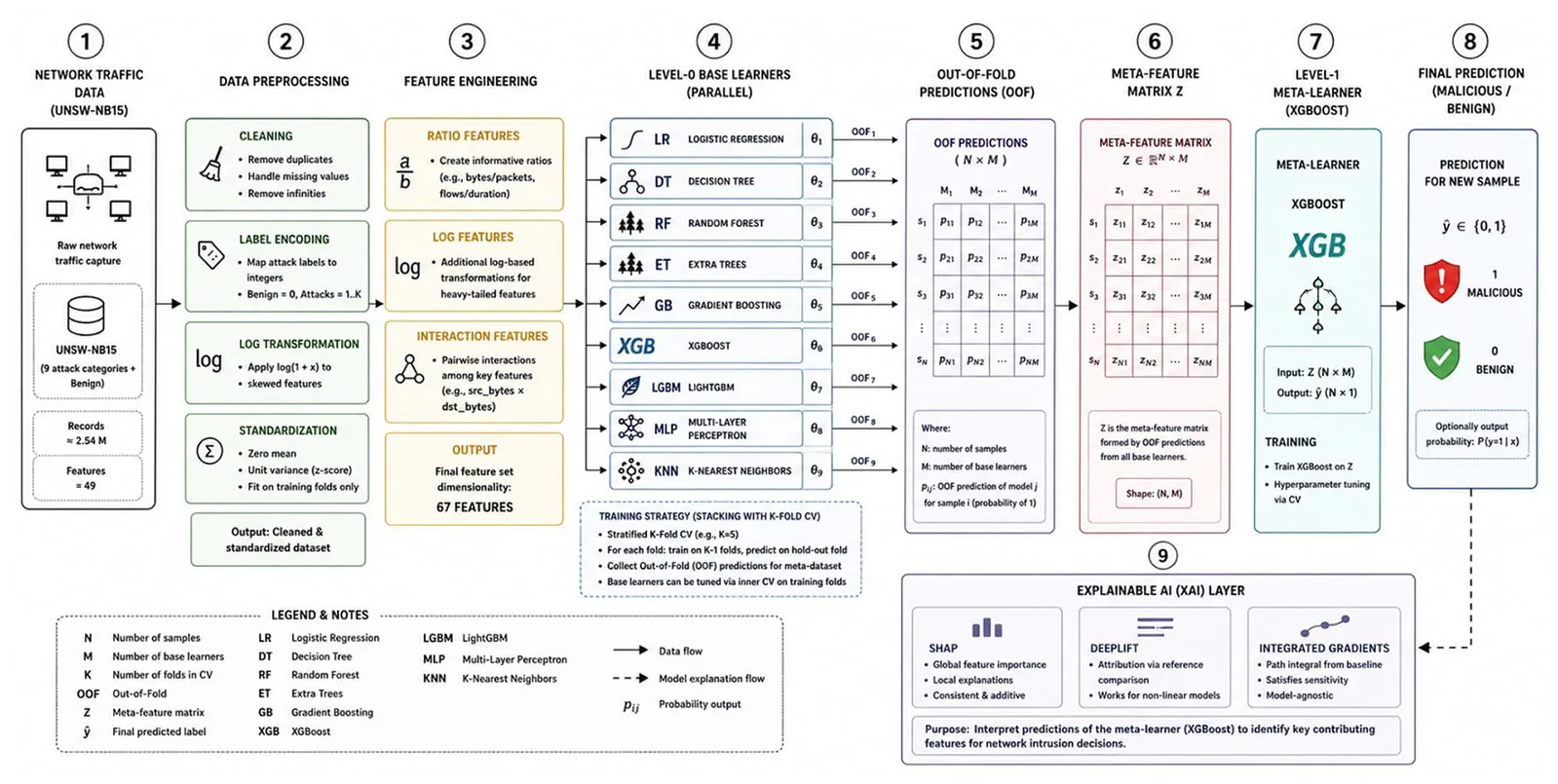
Complete CyberShield Analytics pipeline. Nine sequential stages process raw UNSW-NB15 network traffic from data ingestion through the tri-method XAI layer.

**Figure 10:**
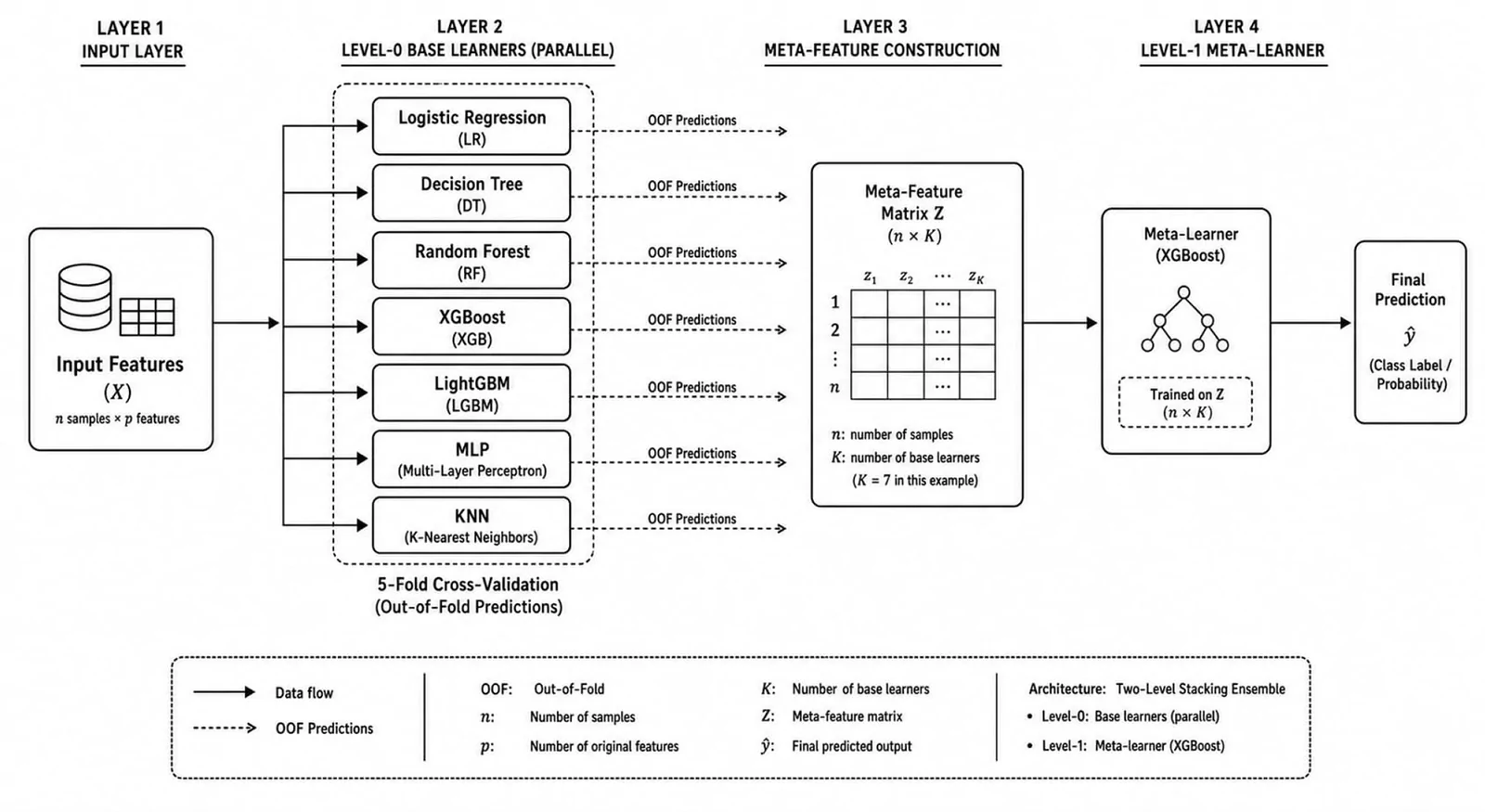
Two-level stacking ensemble architecture.

#### 3.4.1 Level-0 base learners


$$\begin{array}{l} L_{0}=\left\{\text{LR},\text{DT},\text{RF},\text{ET},\text{XGB},\text{LGBM},\text{GB},\text{MLP},\text{KNN}\right\}. \end{array}$$ (12)

Each base learner $h_{k}\in L_{0}$ generates out-of-fold (OOF) class-probability estimates via internal 5-fold cross-validation, constructing the meta-feature matrix **Z** ∈ ℝ^*n*×*K*^:


$$\begin{array}{l} \text{Z}=\left[\begin{array}{cccc} {\hat{p}}_{1}^{\left(1\right)} & {\hat{p}}_{1}^{\left(2\right)} & \cdots & {\hat{p}}_{1}^{\left(K\right)}\\ \vdots & \vdots & \ddots & \vdots \\ {\hat{p}}_{n}^{\left(1\right)} & {\hat{p}}_{n}^{\left(2\right)} & \cdots & {\hat{p}}_{n}^{\left(K\right)} \end{array}\right],\;{\hat{p}}_{i}^{\left(k\right)}=P\left(y_{i}=1|h_{k},{\text{x}}_{i}\right). \end{array}$$ (13)

#### 3.4.2 Level-1 meta-learner

An XGBoost classifier (Chen and Guestrin, 2016) serves as the meta-learner $L_{1}$, trained on **Z**:


$$\begin{array}{l} {ŷ}_{i}=L_{1}\left({\text{Z}}_{i}\right)=\mathrm{XGBoost}\left({\hat{p}}_{i}^{\left(1\right)},{\hat{p}}_{i}^{\left(2\right)},\ldots ,{\hat{p}}_{i}^{\left(K\right)}\right). \end{array}$$ (14)

$$\begin{array}{l} Ŷ=\mathrm{sign}\left(L_{1}\left(\text{Z}\right)-0.5\right). \end{array}$$ (15)

### 3.5 Implementation details and hyperparameters

All models were implemented in Python 3.11 using scikit-learn 1.4 (Pedregosa et al., 2011), XGBoost 2.0 (Chen and Guestrin, 2016), and LightGBM 4.3. Experiments were executed on a workstation with an Intel Core i7 (8 cores, 16 threads) CPU and 32 GB RAM. To ensure reproducibility, a fixed random seed (random_state = 42) was applied to all stochastic components. Table 4 reports the principal hyperparameters of each Level-0 base learner and the Level-1 meta-learner; values were selected by grid search nested within the training folds so that no test data influenced model configuration.

**Table 4:** Principal hyperparameters of the base and meta learners.

Model	Key hyperparameters
Logistic regression	C=1.0, penalty=l2, solver=lbfgs, max_iter=1000, class_weight=balanced
Decision tree	max_depth=20, min_samples_split=10, criterion=gini, class_weight=balanced
Random forest	n_estimators=300, max_depth=None, min_samples_leaf=2, class_weight=balanced, n_jobs=-1
Extra trees	n_estimators=300, max_depth=None, min_samples_leaf=2, class_weight=balanced
Gradient boosting	n_estimators=200, learning_rate=0.1, max_depth=3
XGBoost	n_estimators=400, learning_rate=0.05, max_depth=6, subsample=0.8, colsample_bytree=0.8, scale_pos_weight=0.565
LightGBM	n_estimators=400, learning_rate=0.05, num_leaves=63, subsample=0.8, scale_pos_weight=0.565
MLP	Hidden layers (128, 64), activation=relu, solver=adam, alpha=1e-4, batch_size=256, max_iter=200
KNN	n_neighbors=15, weights=distance, metric=minkowski
Meta-learner (XGBoost)	n_estimators=200, learning_rate=0.05, max_depth=3, subsample=0.9

### 3.6 Evaluation metrics

Performance is quantified using eight complementary metrics Equations 16–22:


$$\begin{array}{l} \text{Accuracy}=\frac{TP+TN}{TP+TN+FP+FN}, \end{array}$$ (16)

$$\begin{array}{l} \text{Precision}=\frac{TP}{TP+FP},\text{ Recall}=\frac{TP}{TP+FN}, \end{array}$$ (17)

$$\begin{array}{l} F_{1}=2\cdot \frac{\text{Precision}\times \text{Recall}}{\text{Precision}+\text{Recall}}, \end{array}$$ (18)

$$\begin{array}{l} \text{AUC-ROC}=\int_{0}^{1}\text{TPR}\left(t\right)d\left[\text{FPR}\left(t\right)\right], \end{array}$$ (19)

$$\begin{array}{l} L_{\log}=-\frac{1}{N}\sum_{i=1}^{N}\left[y_{i}\log{\hat{p}}_{i}+\left(1-y_{i}\right)\log\left(1-{\hat{p}}_{i}\right)\right], \end{array}$$ (20)

$$\begin{array}{l} \text{MAE}=\frac{1}{N}\sum_{i=1}^{N}|y_{i}-{ŷ}_{i}|, \end{array}$$ (21)

$$\begin{array}{l} \text{RMSE}=\sqrt{\frac{1}{N}\sum_{i=1}^{N}{\left(y_{i}-{ŷ}_{i}\right)}^{2}}. \end{array}$$ (22)

### 3.7 5-fold stratified cross-validation

All models were evaluated using 5-Fold Stratified Cross-Validation (SKF-CV), preserving the class distribution *P*(*y* = 1) ≈ 0.639 across each fold (James et al., 2021) (Table 5). The mean and standard deviation of the *F*_1_-score across folds {*s*_1_, *s*_2_, *s*_3_, *s*_4_, *s*_5_} are reported as Equation 23:


$$\begin{array}{l} \bar{s}=\frac{1}{5}\overset{5}{\underset{k=1}{\sum }}s_{k},\;\sigma_{s}=\sqrt{\frac{1}{4}\overset{5}{\underset{k=1}{\sum }}{\left(s_{k}-\bar{s}\right)}^{2}}. \end{array}$$ (23)

**Table 5:** 5-fold stratified cross-validation *F*_1_-score results.

Model	F1	F2	F3	F4	F5	Mean ±Std
Random Forest	0.9483	0.9500	0.9488	0.9506	0.9493	0.9494 ± 0.0008
XGBoost	0.9455	0.9470	0.9485	0.9484	0.9486	0.9476 ± 0.0012
Extra trees	0.9469	0.9491	0.9467	0.9485	0.9474	0.9477 ± 0.0009
LightGBM	0.9422	0.9431	0.9446	0.9464	0.9455	0.9444 ± 0.0015
**Stacking Ens**.	**0.9481**	**0.9491**	**0.9497**	**0.9514**	**0.9513**	**0.9499** **±0.0013**

Bold values indicate the best result for the corresponding metric.

#### 3.7.1 Leakage-free fitting protocol

To guarantee the absence of information leakage, *every* data-driven transformation is fitted strictly on the training portion of each fold and merely applied to the held-out portion. The leakage-free fitting protocol is illustrated in Figure 11. Concretely, within each of the five training folds the following are estimated using training data only: (i) the label-encoding maps for proto, service, and state; (ii) the skewness thresholds and resulting log- transform decisions; (iii) all feature-engineering statistics (ratios, differences, and interaction terms in Section 3.3); (iv) the StandardScaler means and standard deviations (Equation 3); and (v) the base-learner parameters used to generate out-of-fold probability meta-features. Because the entire chain is wrapped in a single sklearn.Pipeline (Pedregosa et al., 2011) and cloned per fold, no statistic derived from a validation fold or from the final held-out test set can influence any fitted quantity. Unseen categorical levels encountered at inference are mapped to a reserved “unknown” code rather than refitting the encoder.

**Figure 11:**
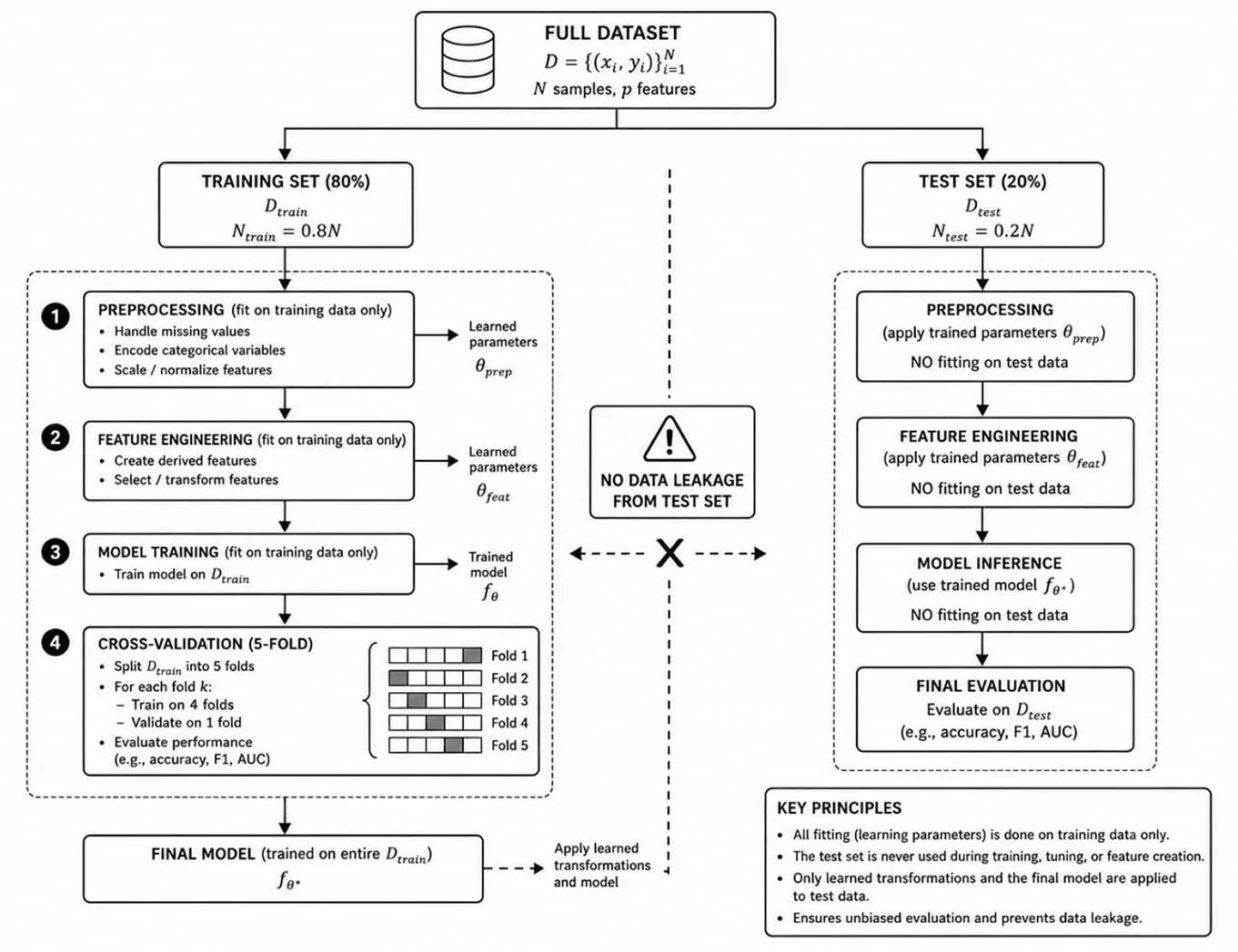
Leakage-free ML evaluation workflow.

### 3.8 Statistical significance testing

Two complementary significance tests were applied to the five fold-wise *F*_1_-score vectors (Demšar, 2006) and Equations 24, 25.

#### 3.8.1 Wilcoxon signed-rank test (non-parametric)


$$\begin{array}{l} W=\underset{i:d_{i}\neq 0}{\sum }\mathrm{sgn}\left(d_{i}\right)\cdot R_{i},\;d_{i}=a_{i}-b_{i}. \end{array}$$ (24)

#### 3.8.2 Paired *t*-test (parametric)


$$\begin{array}{l} t=\frac{\bar{d}}{s_{d}/\sqrt{n}},\;\bar{d}=\frac{1}{n}\overset{n}{\underset{i=1}{\sum }}d_{i},\;s_{d}=\sqrt{\frac{\sum_{i=1}^{n}{\left(d_{i}-\bar{d}\right)}^{2}}{n-1}}, \end{array}$$ (25)

where *n* = 5 folds. Statistical significance was declared at α = 0.05.

### 3.9 Explainable AI framework

#### 3.9.1 SHAP (SHapley Additive exPlanations)


$$\begin{array}{l} \phi_{j}\left(f\right)=\underset{S\subseteq F\backslash \left\{j\right\}}{\sum }\frac{|S|!\left(|F|-|S|-1\right)!}{|F|!}\left[f\left(S\cup \left\{j\right\}\right)-f\left(S\right)\right]. \end{array}$$ (26)

Global feature importance (Lundberg et al., 2020) Equations 26–29:


$$\begin{array}{l} {\bar{\phi }}_{j}=\frac{1}{N}\overset{N}{\underset{i=1}{\sum }}|\phi_{j}^{\left(i\right)}|. \end{array}$$ (27)

#### 3.9.2 DeepLIFT


$$\begin{array}{l} C_{\Delta x_{j}\Delta y}=\frac{\Delta y}{\Delta x_{j}}\cdot \Delta x_{j},\;\Delta x_{j}=x_{j}-x_{j}^{\prime }. \end{array}$$ (28)

#### 3.9.3 Integrated gradients


$$\begin{array}{l} {\text{IG}}_{j}\left(x\right)=\left(x_{j}-x_{j}^{\prime }\right)\times \int_{\alpha =0}^{1}\frac{\partial F\left(x^{\prime }+\alpha \left(x-x^{\prime }\right)\right)}{\partial x_{j}}d\alpha , \end{array}$$ (29)

$$\begin{array}{l} \overset{p}{\underset{j=1}{\sum }}{\text{IG}}_{j}\left(x\right)=F\left(x\right)-F\left(x^{\prime }\right). \end{array}$$ (30)

## 4 Results and analysis

### 4.1 Exploratory data analysis findings

The target variable exhibits a class imbalance ratio of approximately 1.77:1 (malicious: 164,673; benign: 93,000). Among attack categories, **Generic** attacks constitute the largest malicious class (58,871 samples, 35.7% of all attacks), followed by Exploits (44,525) and Fuzzers (24,246), while Worms represent the rarest category (174 samples).

### 4.2 5-fold stratified cross-validation performance

Table 5 reports the fold-wise F1-scores obtained under 5-fold stratified cross-validation, and Figure 12 summarizes the corresponding mean performance and per-fold accuracy trajectories. The stacking ensemble attains the highest mean F1-score (0.9499 ± 0.0013), followed by Random Forest (0.9494 ± 0.0008), Extra Trees (0.9477 ± 0.0009), XGBoost (0.9476 ± 0.0012), and LightGBM (0.9444 ± 0.0015). All standard deviations are below 0.0016, indicating stable behaviour across partitions. Notably, the ensemble achieves the highest F1-score in four of the five folds and is never the worst-performing model in any fold, whereas LightGBM is the weakest learner in every fold. The narrow spread across models—a range of 0.0055 in mean F1 between the best and worst candidate—confirms that the UNSW-NB15 binary task is close to saturation for modern tree-based learners, and that the ensemble's advantage lies in consistency rather than magnitude.

**Figure 12:**
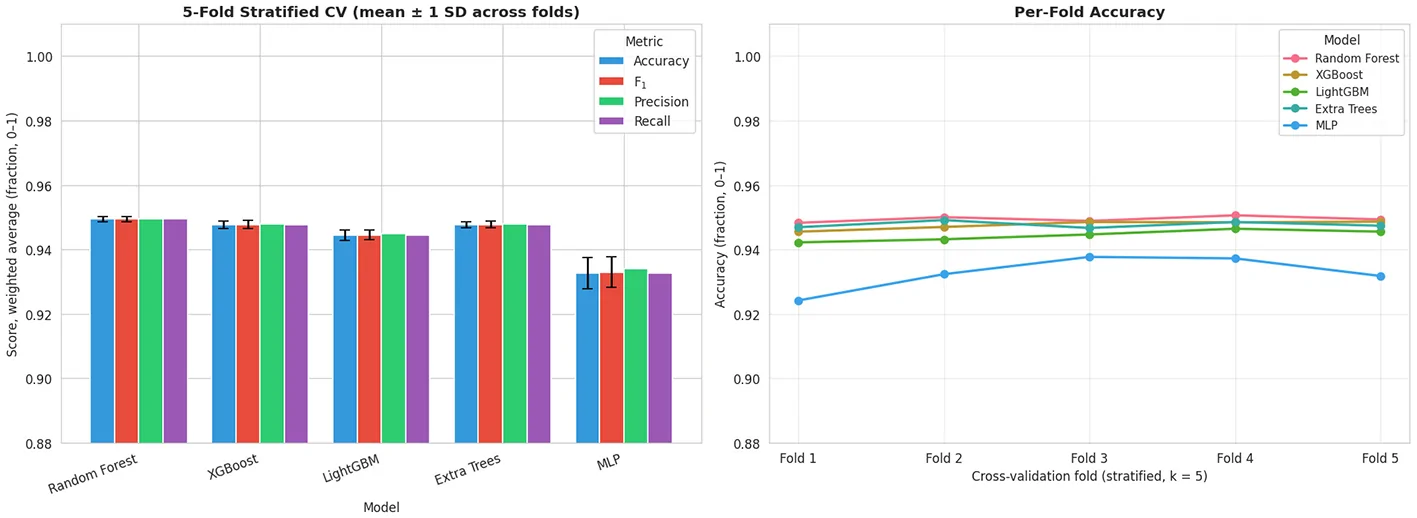
5-fold CV results. **Left**: Mean ± std of Accuracy, F1, Precision, Recall. **Right**: Per-fold accuracy trajectories.

### 4.3 Test set performance comparison

Table 6 reports the complete performance of all ten evaluated models on the fully held-out test set of 51,535 instances. The stacking ensemble achieves the best Accuracy, Precision, Recall, and F1-score (all 0.9535) and the highest AUC-ROC (0.9929), outperforming every individual base learner. Random Forest is the strongest single model (Accuracy = 0.9528, AUC-ROC = 0.9923), followed by Extra Trees (0.9504), XGBoost (0.9491), and LightGBM (0.9463). Logistic Regression is the weakest learner (Accuracy = 0.8650, F1 = 0.8618), reflecting the non-linear separability of the feature space and confirming the necessity of non-linear decision boundaries for this task. Figure 13 presents the ROC curves for all models together with the precision-recall curve of the ensemble, and Figure 14 compares base-classifier performance across the six principal metrics. Figure 15 shows the confusion matrices for the ensemble: of 18,600 Normal flows, 17,429 are correctly classified and 1,171 are false positives (true negative rate 0.9370), while of 32,935 Attack flows, 31,708 are correctly detected and 1,227 are missed (recall 0.9627). The higher recall on the Attack class than on the Normal class is operationally desirable in an intrusion-detection setting, where the cost of a missed intrusion typically exceeds that of a false alarm.

**Table 6:** Complete model performance on held-out test set (*n* = 51, 535).

Model	Acc.	Prec.	Rec.	*F*_1_	AUC	LL	MAE	RMSE
⋆**Stacking**	**0.9535**	**0.9535**	**0.9535**	**0.9535**	**0.9929**	0.1132	**0.0465**	**0.2157**
Random Forest	0.9528	0.9529	0.9528	0.9529	0.9923	0.1132	0.0472	0.2172
Extra trees	0.9504	0.9504	0.9504	0.9504	0.9902	0.1697	0.0496	0.2228
XGBoost	0.9491	0.9494	0.9491	0.9492	0.9919	**0.1069**	0.0509	0.2257
LightGBM	0.9463	0.9467	0.9463	0.9464	0.9911	0.1129	0.0537	0.2318
Decision tree	0.9397	0.9397	0.9397	0.9397	0.9367	2.0882	0.0604	0.2457
Grad. boosting	0.9345	0.9344	0.9345	0.9345	0.9873	0.1405	0.0655	0.2558
MLP	0.9331	0.9347	0.9331	0.9335	0.9859	0.1389	0.0669	0.2587
KNN	0.9278	0.9281	0.9278	0.9279	0.9760	0.5015	0.0722	0.2686
Logistic Reg.	0.8650	0.8662	0.8650	0.8618	0.9294	0.3092	0.1350	0.3674

Acc, Accuracy; Prec, Precision; Rec, Recall; LL, Log-Loss; AUC, AUC-ROC.

Acc Bold: best per metric; ⋆: proposed model.

**Figure 13:**
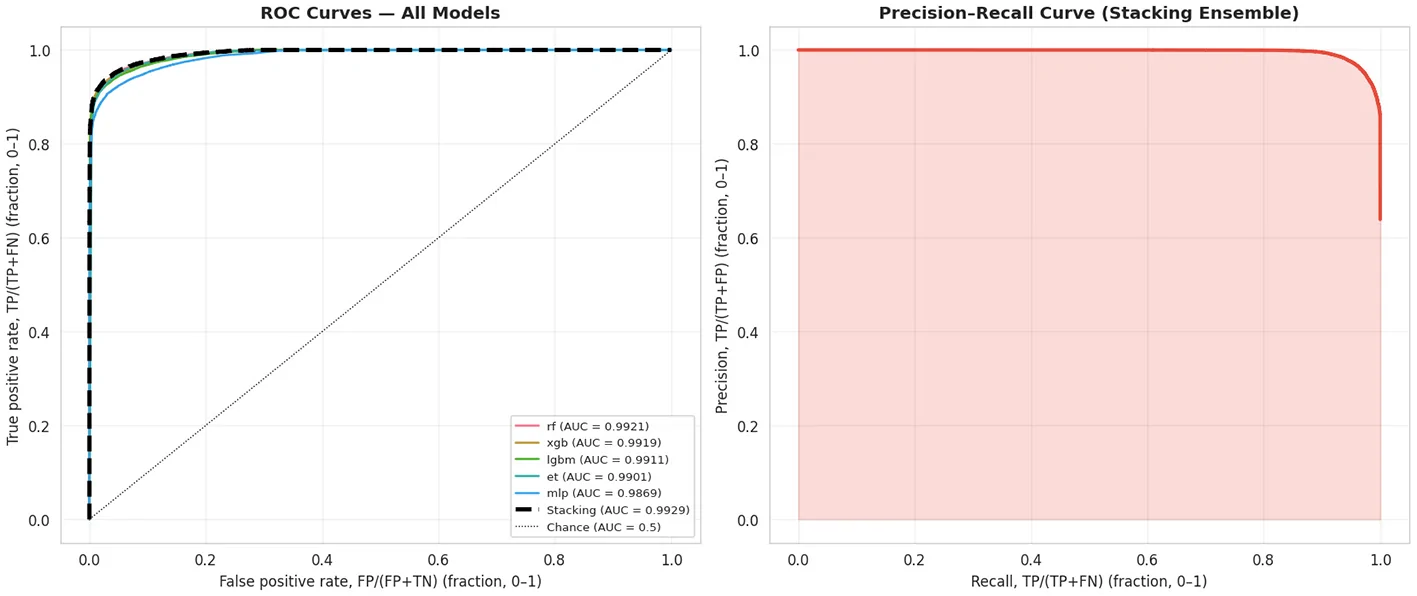
**Left**: ROC curves for all evaluated models (Stacking Ensemble AUC = 0.9929). **Right**: Precision-Recall curve for the Stacking Ensemble.

**Figure 14:**
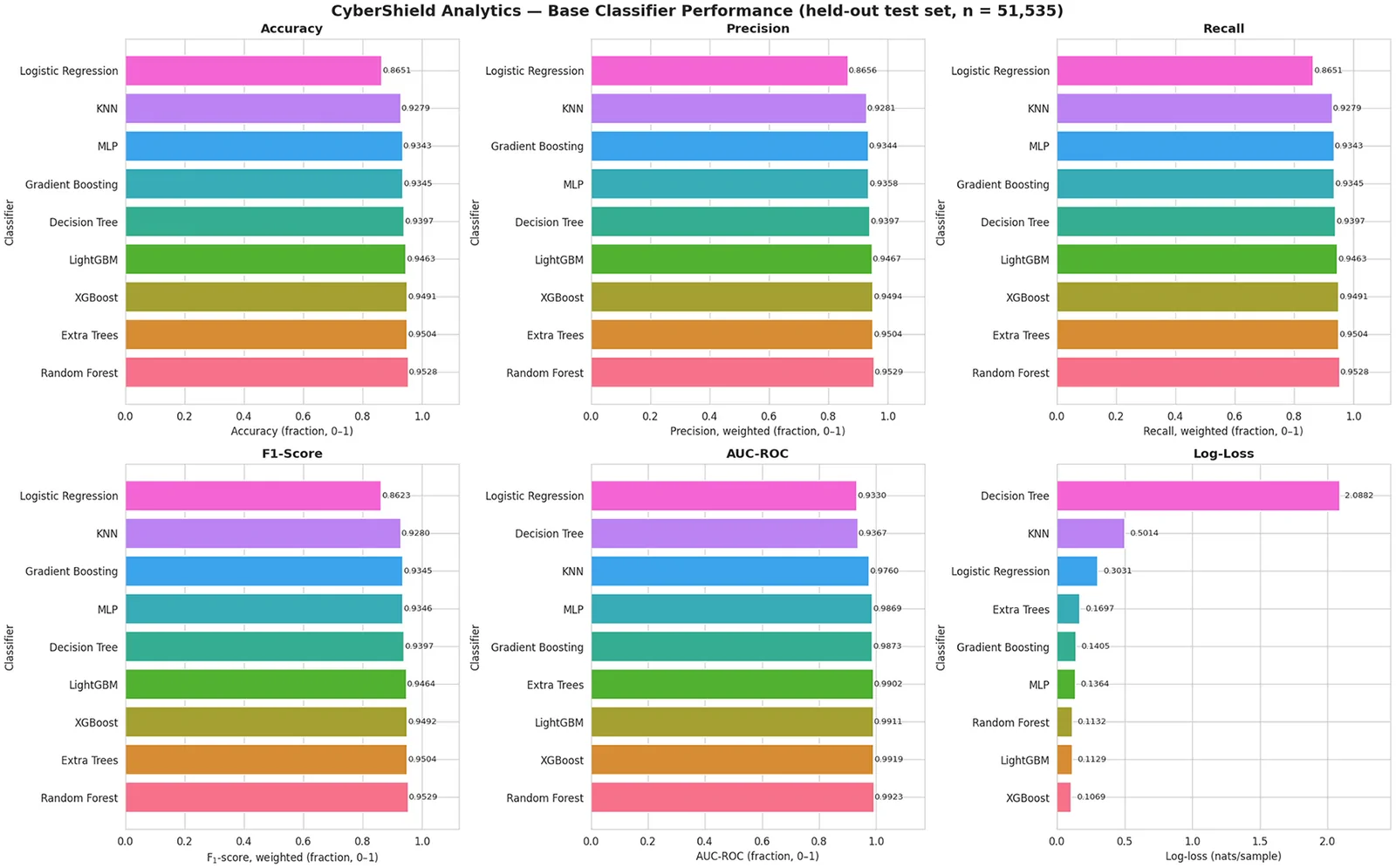
Base classifier performance comparison on the held-out test set.

**Figure 15:**
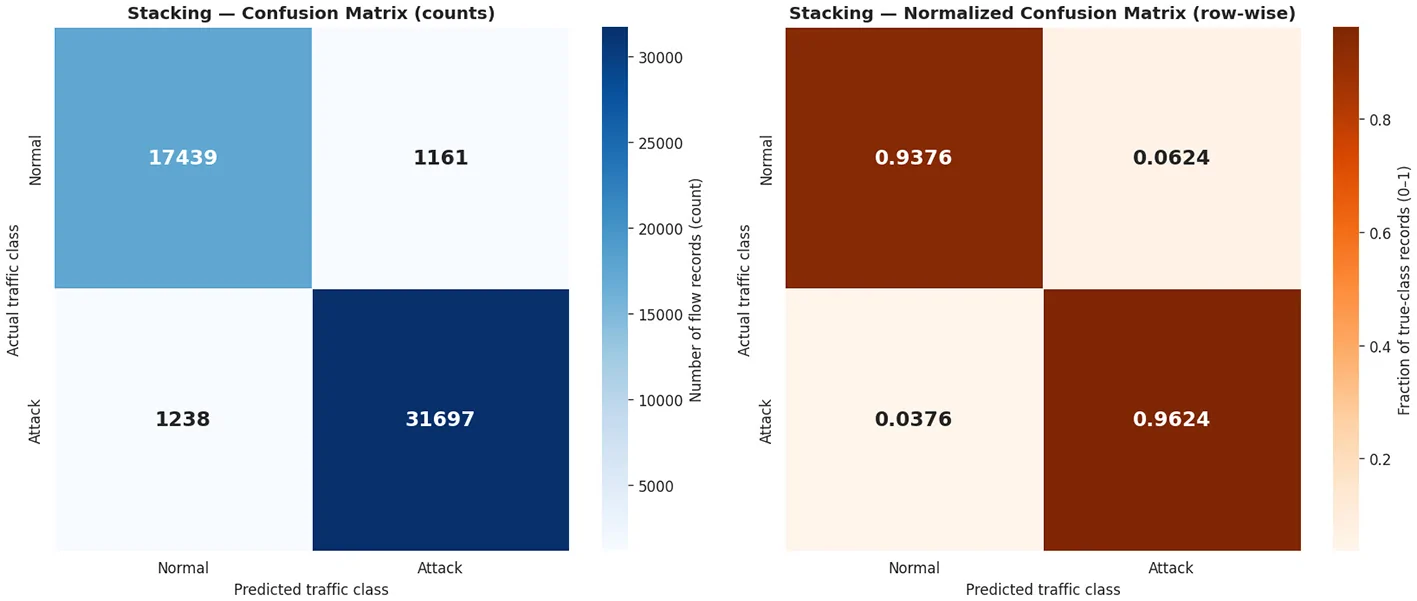
Confusion matrices for the Stacking Ensemble on the held-out test set.

### 4.4 Error evaluation analysis

Table 7 decomposes the prediction error of each model, and Figure 16 presents the corresponding comparison across MAE, MSE, RMSE, and log-loss. The stacking ensemble attains the lowest MAE (0.04653) and RMSE (0.21571) of all evaluated models, marginally ahead of Random Forest (MAE 0.04715, RMSE 0.21715) and ahead of XGBoost (MAE 0.05092, RMSE 0.22565) and LightGBM (MAE 0.05373, RMSE 0.23180). Logistic Regression records the highest MAE (0.13496) and RMSE (0.36736), consistent with its accuracy deficit in Table 6. The log-loss ordering differs from the ordering of the threshold-based metrics: XGBoost achieves the lowest log-loss (0.10686), slightly below the ensemble (0.11318) and Random Forest (0.11320), indicating that the single gradient-boosted model produces marginally sharper probability estimates even though it misclassifies more instances. The Decision Tree is a clear outlier, with a log-loss of 2.08824 despite an accuracy of 0.9397; this reflects the near-deterministic leaf probabilities characteristic of unregularized trees, which incur severe penalties on confidently incorrect predictions. K-Nearest Neighbors shows the same pathology to a lesser degree (log-loss 0.50147). These results indicate that accuracy alone is an insufficient basis for model selection when calibrated probabilities are required for risk-ranked alert triage.

**Table 7:** Error metric decomposition on the held-out test set.

Model	MAE	MSE	RMSE	Log-Loss
⋆ **Stacking**	**0.04653**	**0.04653**	**0.21571**	0.11318
Random forest	0.04715	0.04715	0.21715	0.11320
Extra trees	0.04962	0.04962	0.22275	0.16965
XGBoost	0.05092	0.05092	0.22565	**0.10686**
LightGBM	0.05373	0.05373	0.23180	0.11294
Gradient boosting	0.06545	0.06545	0.25583	0.14047
MLP	0.06691	0.06691	0.25866	0.13891
Decision tree	0.06035	0.06035	0.24566	2.08824
KNN	0.07216	0.07216	0.26863	0.50147
Logistic reg.	0.13496	0.13496	0.36736	0.30925

Bold values indicate the best result for the corresponding metric.

**Figure 16:**
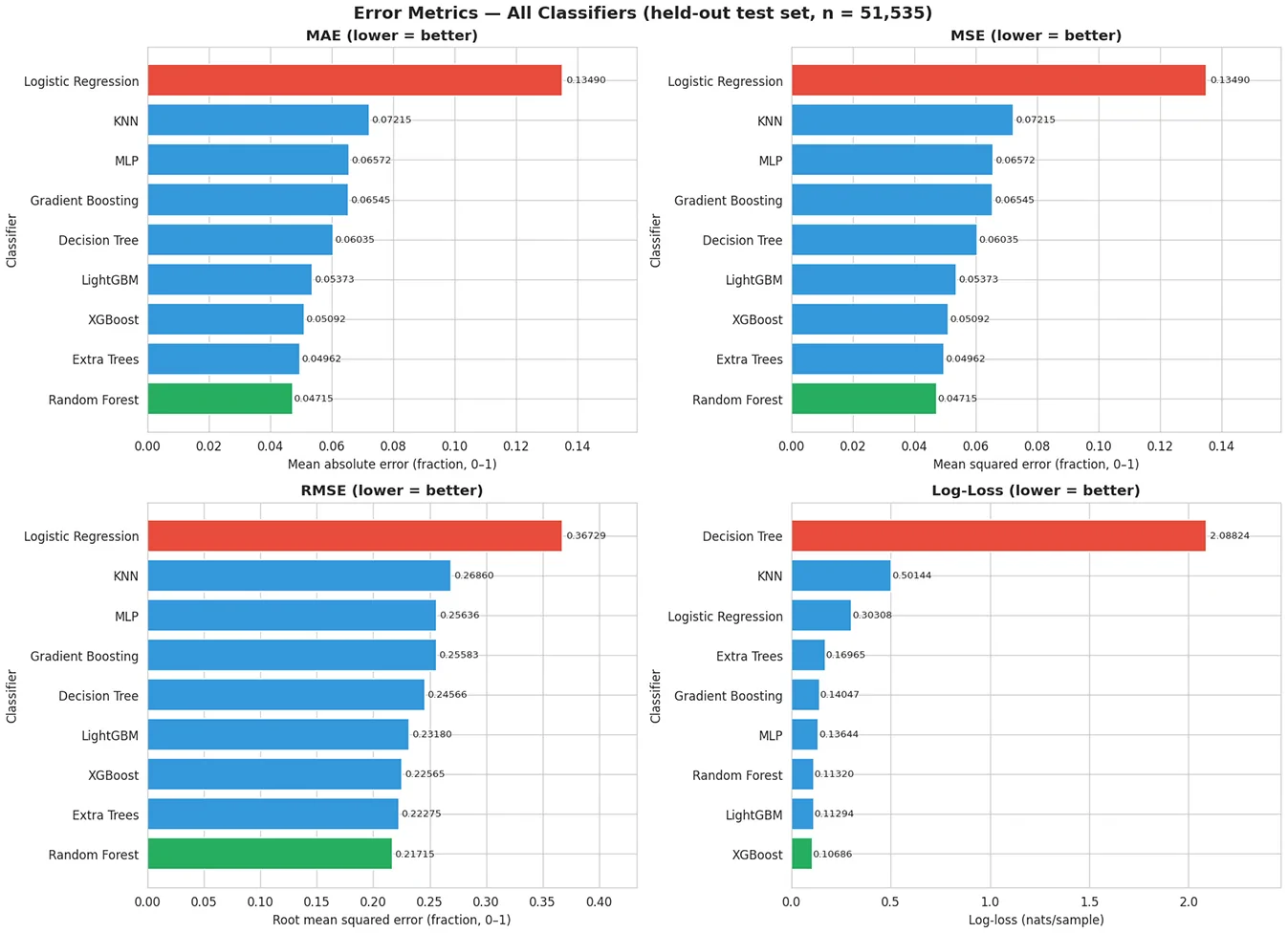
Error metric decomposition across all classifiers.

### 4.5 Statistical significance analysis

Table 8 reports the paired significance tests comparing the stacking ensemble with the four strongest base learners across the five cross-validation folds, and Figure 17 presents the corresponding fold-wise accuracy distributions together with the Wilcoxon and paired *t*-test *p*-values. The ensemble attains a mean F1-score of 0.9499 in every comparison. Under the paired *t*-test the improvement is significant against LightGBM (ΔF1 = +0.0055, *t* = 25.108, *p* < 0.0001), XGBoost (ΔF1 = +0.0023, *t* = 7.165, *p* = 0.0020) and Extra Trees (ΔF1 = +0.0022, *t* = 3.178, *p* = 0.0336), but not against Random Forest (ΔF1 = +0.0005, *t* = 1.090, *p* = 0.3372). The Wilcoxon signed-rank test, which makes no distributional assumption, returns *W* = 0.000 and *p* = 0.0625 for those same three comparisons-the smallest two-sided p-value attainable with five paired observations-and *W* = 5.000, *p* = 0.6250 for Random Forest. The two tests therefore agree on the ordering of the comparisons, and both indicate that the margin over Random Forest lies within fold-to-fold variation. The following subsections examine the reliability of the five-fold protocol and the practical significance of the improvement.

**Table 8:** Statistical significance tests: stacking ensemble vs. baselines (α = 0.05, *n* = 5 folds).

Baseline	${\bar{F}}_{1}^{\text{stack}}$	${\bar{F}}_{1}^{\text{base}}$	*ΔF*_1_	*W*	*p*_*W*_	*t*-stat	*p*_*t*_
Random forest	0.9499	0.9494	+0.0005	5.000	0.6250	1.090	0.3372
XGBoost	0.9499	0.9476	+0.0023	0.000	0.0625	7.165	**0.0020**
LightGBM	0.9499	0.9444	+0.0055	0.000	0.0625	25.108	**< 0.0001**
Extra trees	0.9499	0.9477	+0.0022	0.000	0.0625	3.178	**0.0336**

*W*, Wilcoxon statistic; Bold *p*_*t*_: significant at α = 0.05. Bold values indicate the best result for the corresponding metric.

**Figure 17:**
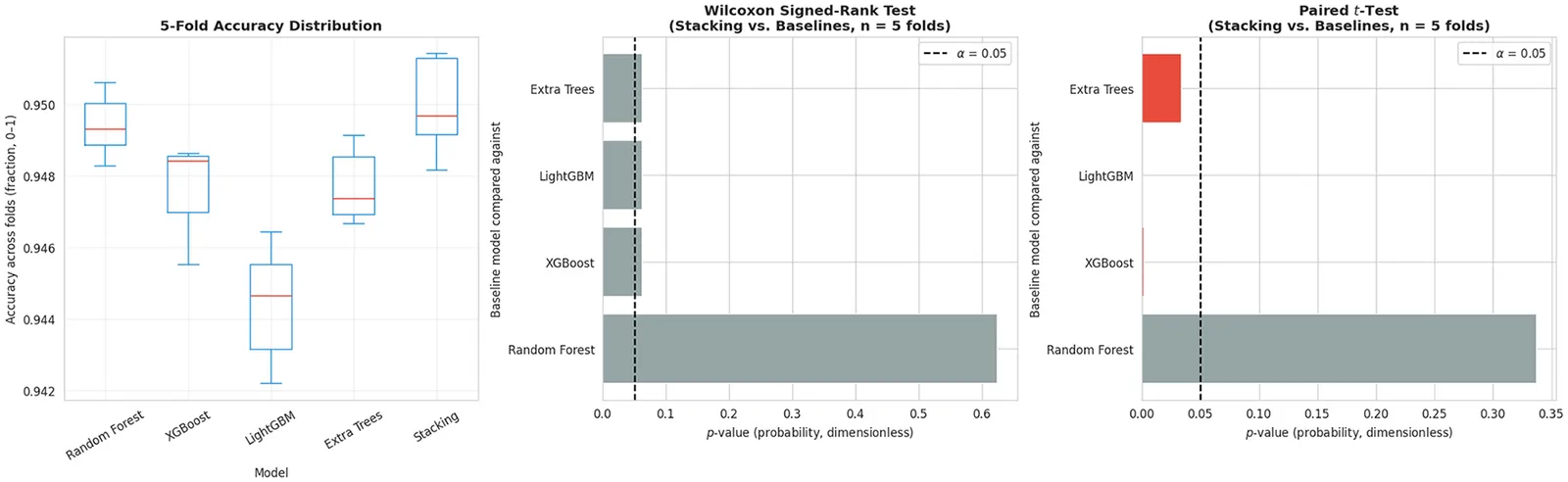
Statistical significance analysis. **Left**: 5-fold accuracy boxplots. **Center**: Wilcoxon *p*-values. **Right**: Paired *t*-test *p*-values.

#### 4.5.1 On the reliability of 5-fold testing

With *n* = 5 paired observations the Wilcoxon Signed-Rank Test is severely power-limited: its smallest attainable two-sided *p*-value is 0.0625, which lies above α = 0.05 and therefore *cannot* reach significance regardless of how consistently the ensemble wins. This is a structural property of the test at *n* = 5, not evidence of an absent effect; indeed the ensemble outperformed XGBoost, LightGBM, and Extra Trees on all five folds (*W* = 0). For this reason the paired *t*-test is treated as the primary inferential instrument, and it confirms significance against three of the four baselines. We nevertheless regard the five-fold design as a limitation (Section 6); repeated stratified cross-validation (e.g., 5 × 10) or the corrected resampled *t*-test would yield more reliable estimates and is recommended for confirmatory studies.

#### 4.5.2 Practical significance of the improvement

The absolute *F*_1_ gain over the strongest baselines is modest (+0.0005 over Random Forest to +0.0055 over LightGBM), and we do not overstate it. Its operational value, however, scales with traffic volume: at a representative enterprise rate of 10^7^ flows per day, the test-set error reduction from XGBoost (MAE 0.0509) to the ensemble (MAE 0.0465) corresponds to roughly 4.4 × 10^4^ fewer misclassified flows per day, a material reduction in analyst alert burden. Equally important, the ensemble attains the highest AUC-ROC (0.9929) and the lowest MAE/RMSE of all models (see Section 4.7), which directly benefits risk-ranked alert triage. The contribution should thus be read as improved robustness, calibration, and statistical reliability at comparable accuracy, rather than a large headline accuracy jump.

### 4.6 SHAP feature importance analysis

Global SHAP importances for the Random Forest base learner are presented in Figure 18, with the corresponding beeswarm summary in Figure 19 and dependence plots for the three highest-ranked features in Figure 20. Representative local explanations for an Attack-predicted and a Normal-predicted instance are given in Figures 21 and 22, respectively, and the pairwise correlation structure of the SHAP values for the top-15 features is shown in Figure 23 and Table 9.

**Figure 18:**
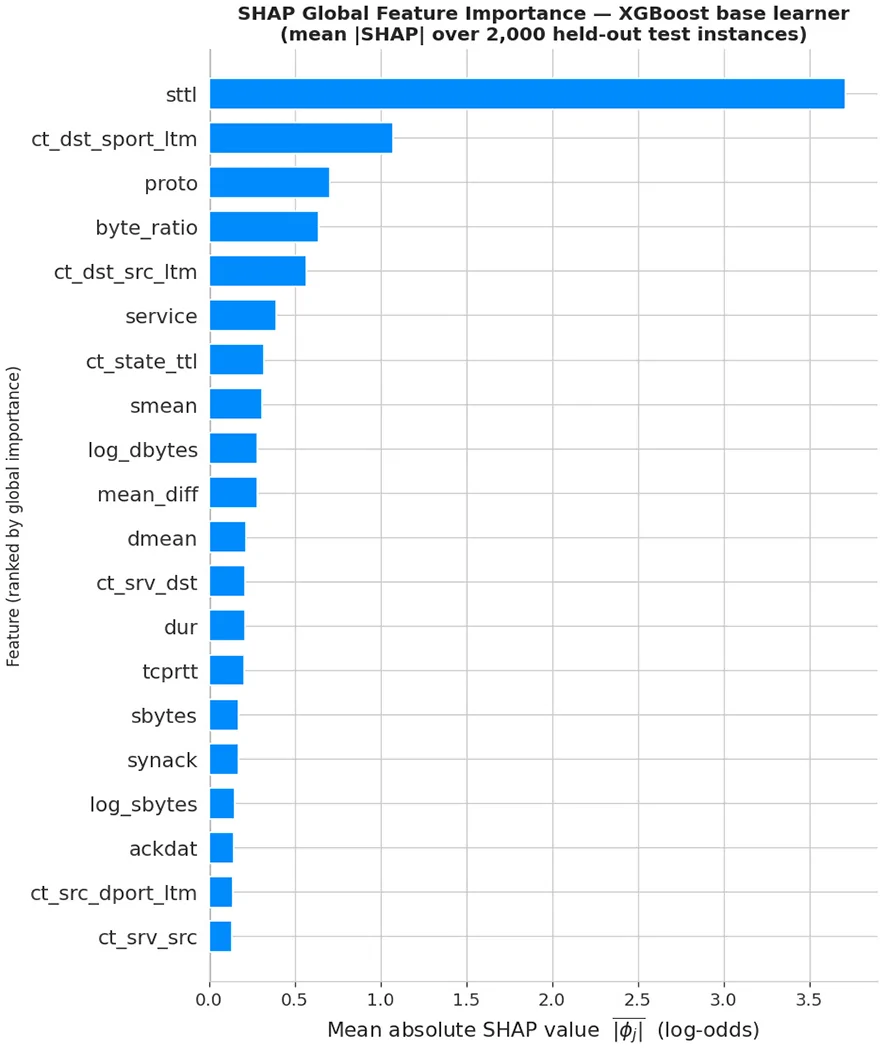
Global SHAP feature importance (mean |ϕ_*j*_|) for the Random Forest base learner on 500 held-out instances.

**Figure 19:**
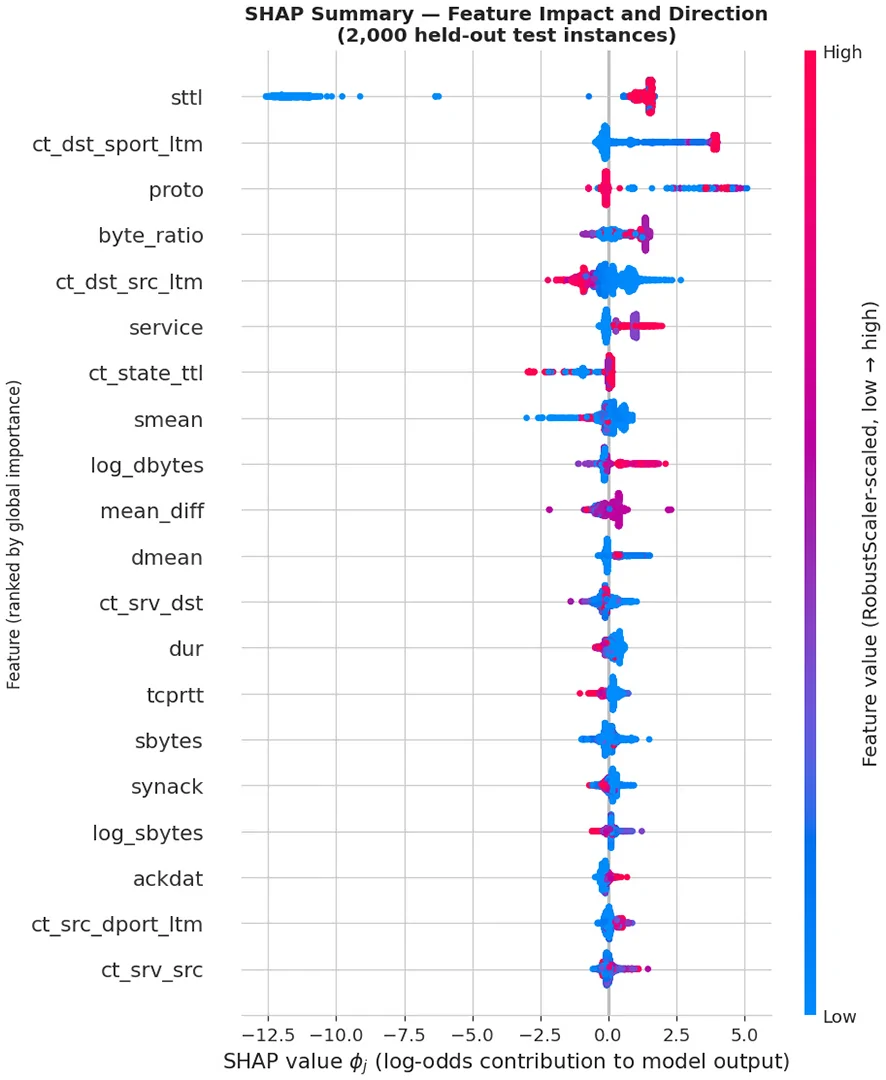
SHAP beeswarm plot showing feature impact direction and magnitude across 500 test instances.

**Figure 20:**
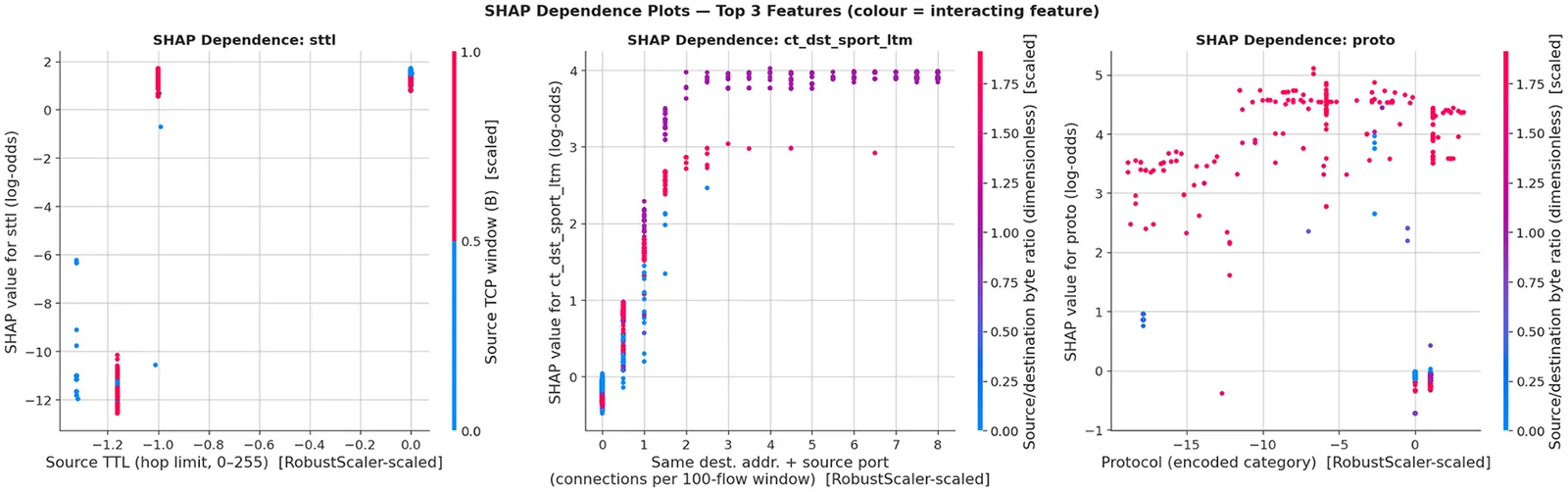
SHAP dependence plots for the three highest-importance features.

**Figure 21:**
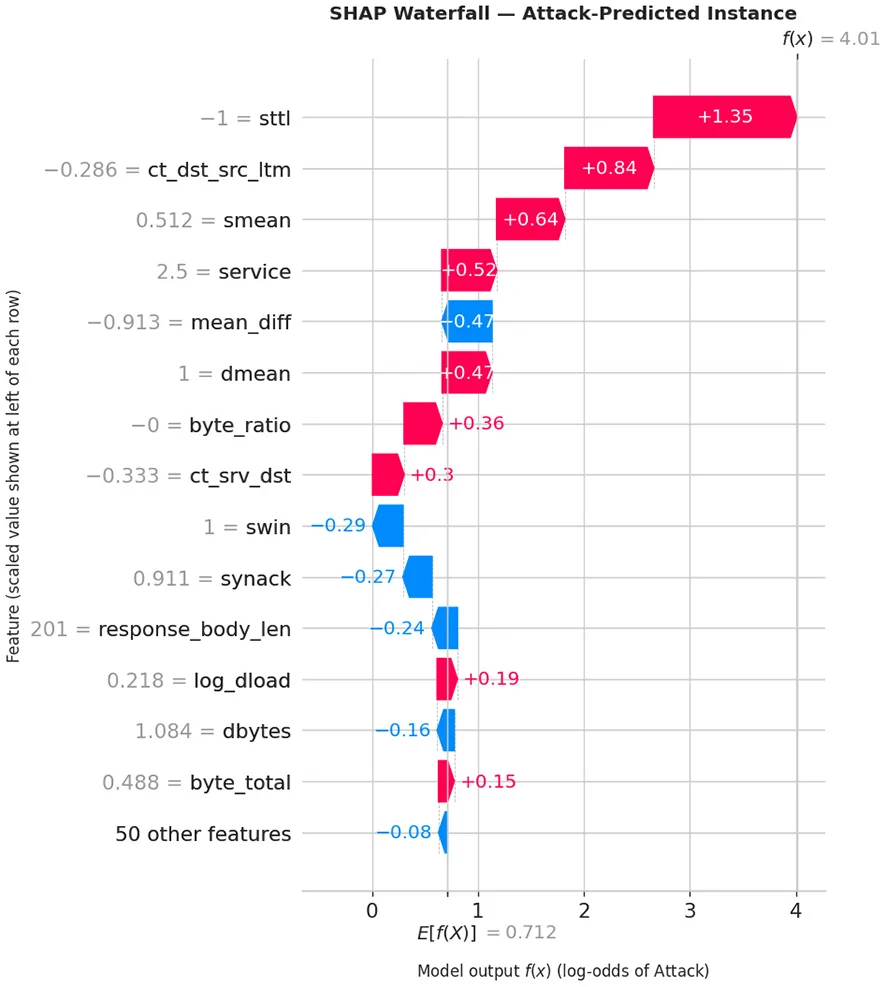
SHAP waterfall plot for a representative Attack-predicted test instance.

**Figure 22:**
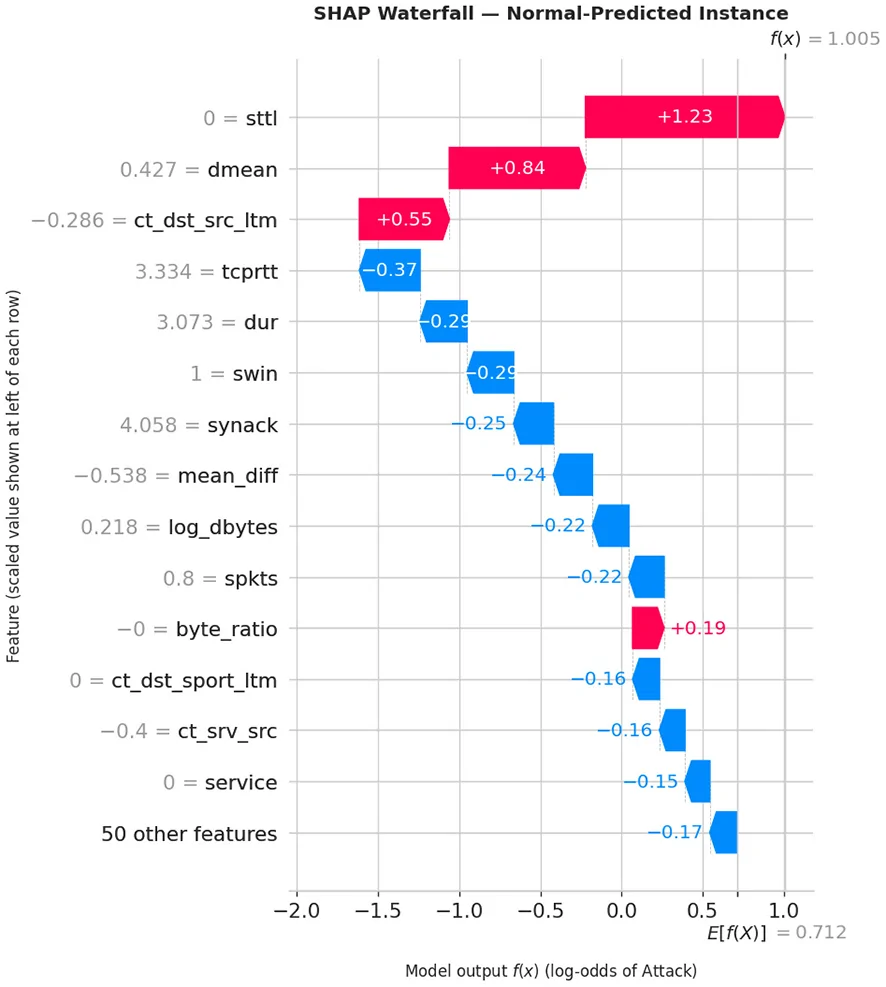
SHAP waterfall plot for a representative Normal-predicted test instance.

**Figure 23:**
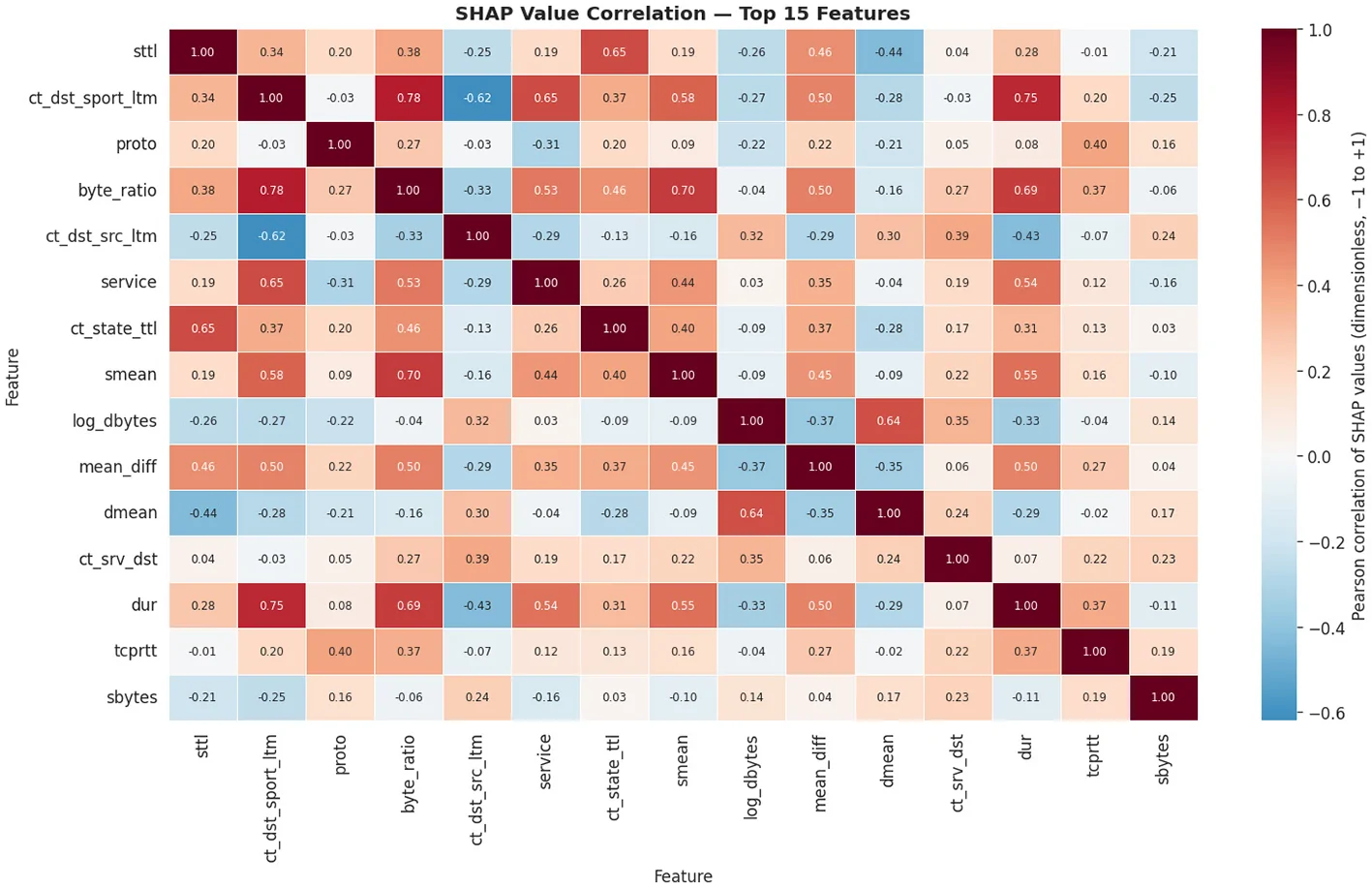
SHAP value correlation matrix for the top 15 features.

**Table 9:** Top-15 SHAP global feature importances (mean |ϕ_*j*_|).

Rank	Feature	${\bar{\phi }}_{j}$	Threat interpretation
1	sttl	3.7122	Source TTL discriminates routing hops for spoofed traffic
2	ct_dst_sport_ltm	1.0752	Destination-port count detects port scanning
3	proto	0.7017	UDP/ICMP overrepresentation signals DoS/DDoS
4	byte_ratio ^†^	0.6397	Payload asymmetry indicates data exfiltration
5	ct_dst_src_ltm	0.5671	Src-dst pair count flags lateral movement
6	service	0.3934	Exploited protocol vulnerabilities
7	ct_state_ttl	0.3209	Anomalous session state combinations
8	smean	0.3108	Abnormal source packet size in shellcode injection
9	log_dbytes ^†^	0.2797	Normalized destination payload volume
10	mean_diff ^†^	0.2792	Directional traffic asymmetry
11	dmean	0.2152	Destination packet size distribution
12	ct_srv_dst	0.2123	Service-per-destination scanning indicator
13	dur	0.2120	Zero-duration flows indicate Fuzzers/DoS
14	tcprtt	0.2050	Elevated RTT in geographically distributed botnets
15	sbytes	0.1738	Raw source payload volume

^†^Engineered feature (see Section 3.3).

#### 4.6.1 Scope of the explainability analysis

The XAI layer explains *component* base learners rather than the meta-learner directly (Thacharodi et al., 2024), and we state this explicitly to avoid over-interpretation. The XGBoost meta-learner operates on the nine-dimensional vector of base-learner probabilities (Equation 14), not on the 67 input features; a SHAP attribution computed on the meta-learner would therefore rank *base learners*, not network-flow features, and would not be actionable for an analyst. To obtain feature-level attributions we apply SHAP to the Random Forest base learner—chosen as a high-performing, natively tree-explainable Level-0 model—and DeepLIFT/Integrated Gradients to the MLP base learner. Because all base learners are trained on the same 67-feature representation, the consensus of independent attribution methods across two different model families is used as a proxy for the features the ensemble collectively relies upon. This consensus-based reading is a deliberate design choice and a recognized limitation: it characterizes the inputs that drive the strongest base learners rather than the meta-combination itself.

### 4.7 DeepLIFT and integrated gradients analysis

The neural network base learner (MLP; Accuracy = 0.9331) was subjected to DeepLIFT (Shrikumar et al., 2017) and Integrated Gradients (Sundararajan et al., 2017) analysis using a zero-vector baseline *x*′ = **0** (Equation 30, 31).

Both methods converged on a consistent set of top-contributing features: sttl, ct_dst_sport_ltm, and byte_ratio appear in the top-5 rankings of all three XAI methods (Capuano et al., 2022).

The Completeness property of Integrated Gradients was numerically verified:


$$\begin{array}{l} \overset{67}{\underset{j=1}{\sum }}{\text{IG}}_{j}\left(x^{\left(0\right)}\right)=F\left(x^{\left(0\right)}\right)-F\left(0\right)\approx 0.9948-0.4998=0.4950. \end{array}$$ (31)

Figure 24 shows the XGBoost learning curve, in which training and cross-validation accuracy converge as the training set grows, indicating that the model is not over-fitted at the operating sample size. Figure 25 compares the probability calibration of XGBoost, Random Forest, Logistic Regression, and the stacking ensemble. Figure 26 compares the top-20 feature rankings produced by Random Forest impurity importance, XGBoost gain importance, and SHAP; sttl is ranked first by all three, confirming cross-method attribution stability.

**Figure 24:**
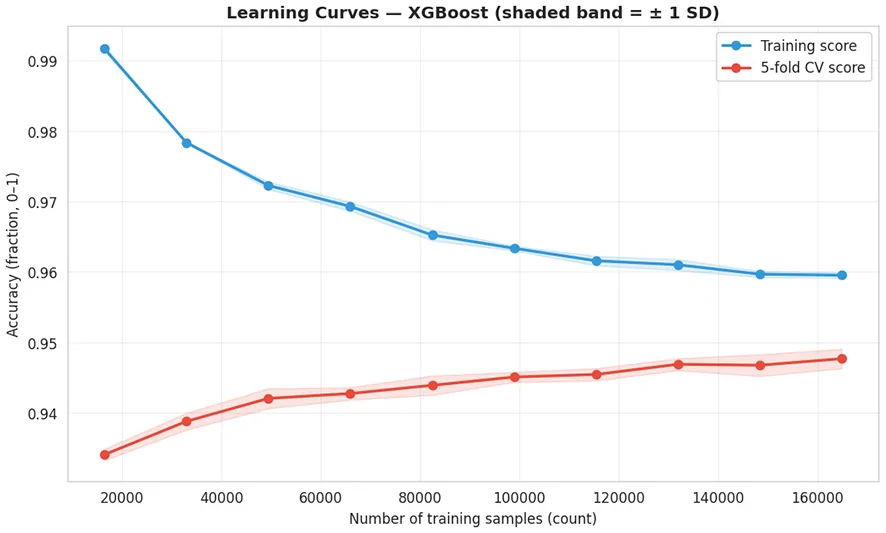
XGBoost learning curve: training accuracy (blue) and 5-fold CV accuracy (red) vs. training set size.

**Figure 25:**
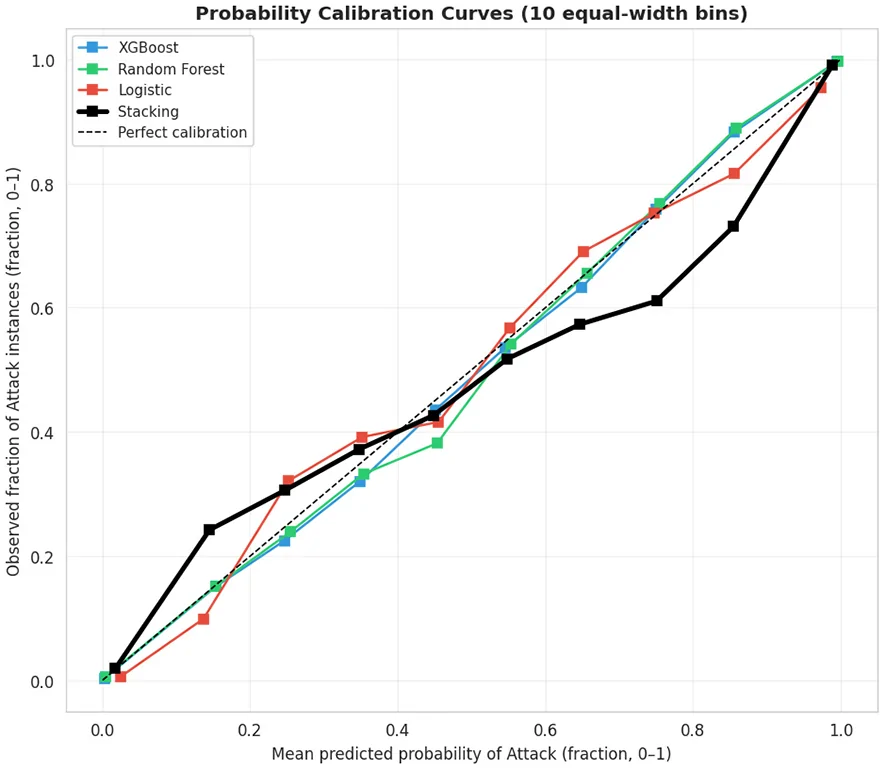
Probability calibration curves for XGBoost, Random Forest, Logistic Regression, and the Stacking Ensemble.

**Figure 26:**
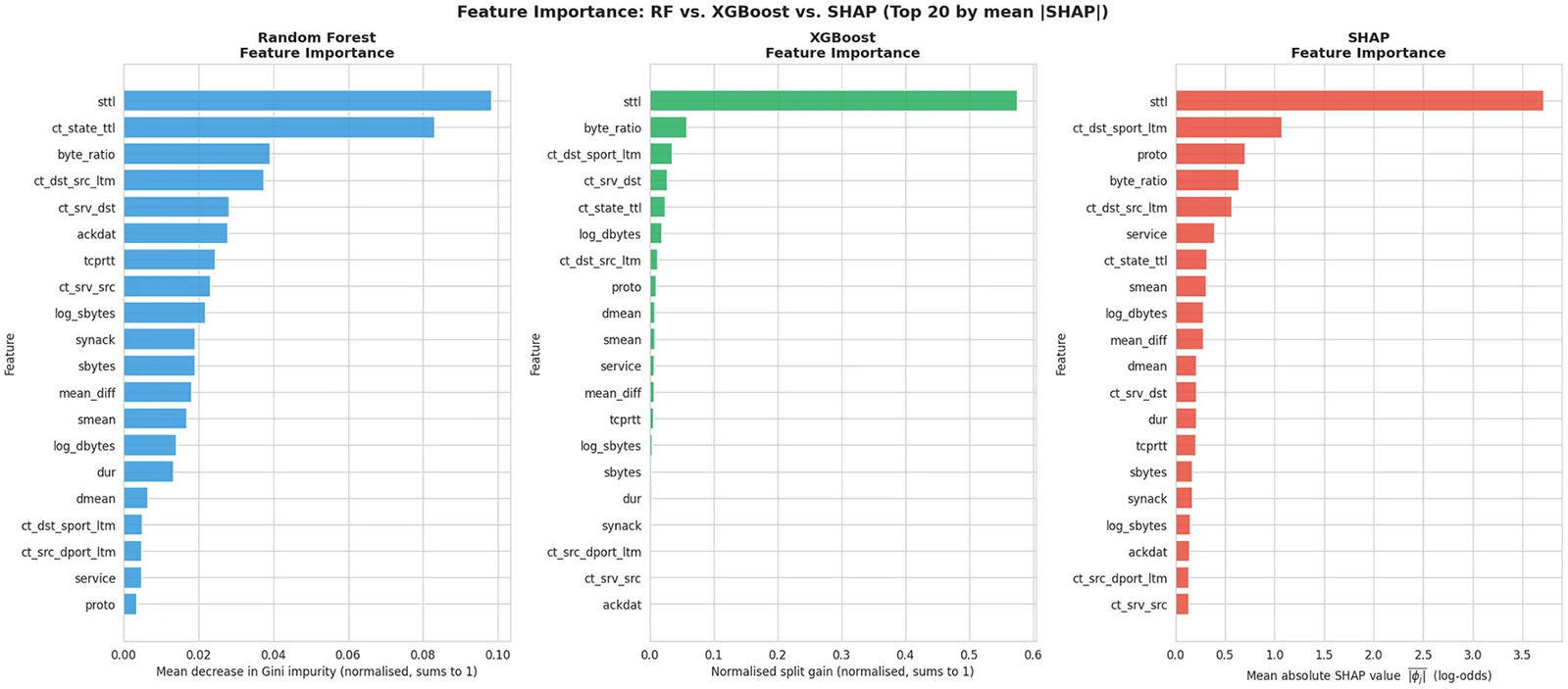
Comparative feature importance rankings (top 20) across Random Forest, XGBoost, and SHAP.

### 4.8 Comparison with state-of-the-art

Table 10 positions CyberShield Analytics relative to previously published results on UNSW-NB15 (Wang et al., 2024, 2025). Kasongo and Sun (2020) report 93.62% accuracy using Random Forest with wrapper-based feature selection. The proposed framework achieves 95.35% accuracy, *F*_1_ = 0.9535, and AUC-ROC = 0.9929 on an 80/20 stratified partition. We emphasize that these figures are not directly comparable: the cited studies differ in task formulation (binary versus multiclass), partitioning protocol, feature-selection procedure, and—critically—in whether preprocessing was fitted before or after the train/test split. Several report a single point estimate on one split without confidence intervals or significance testing, and none reports the F1 and AUC-ROC values needed for a like-for-like comparison under class imbalance. Accordingly, we do not claim a new accuracy record. The distinguishing contribution of CyberShield is that its reported figures are obtained under a strictly leakage-free, pipeline-encapsulated protocol, are accompanied by fold-wise variance and formal significance testing, and are supported by a tri-method explanation layer—properties that none of the compared studies provides jointly (Table 1). Figure 27 presents the Detection Error Tradeoff curves for XGBoost, Random Forest, and the ensemble, and Figure 28 summarizes the relative performance of the four strongest models across five metrics.

**Table 10:** Comparison with state-of-the-art methods on UNSW-NB15.

Method	Year	Accuracy	*F*_1_	AUC	Partition
Kasongo & Sun (RF+FS) (Kasongo and Sun, 2020)	2020	0.9362	—	—	Standard
**CyberShield (Ours)**	**2026**	**0.9535**	**0.9535**	**0.9929**	80/20 Stratified

—, metric not reported; ET, Extra Trees; FS, Feature Selection. Bold values indicate the best result for the corresponding metric.

**Figure 27:**
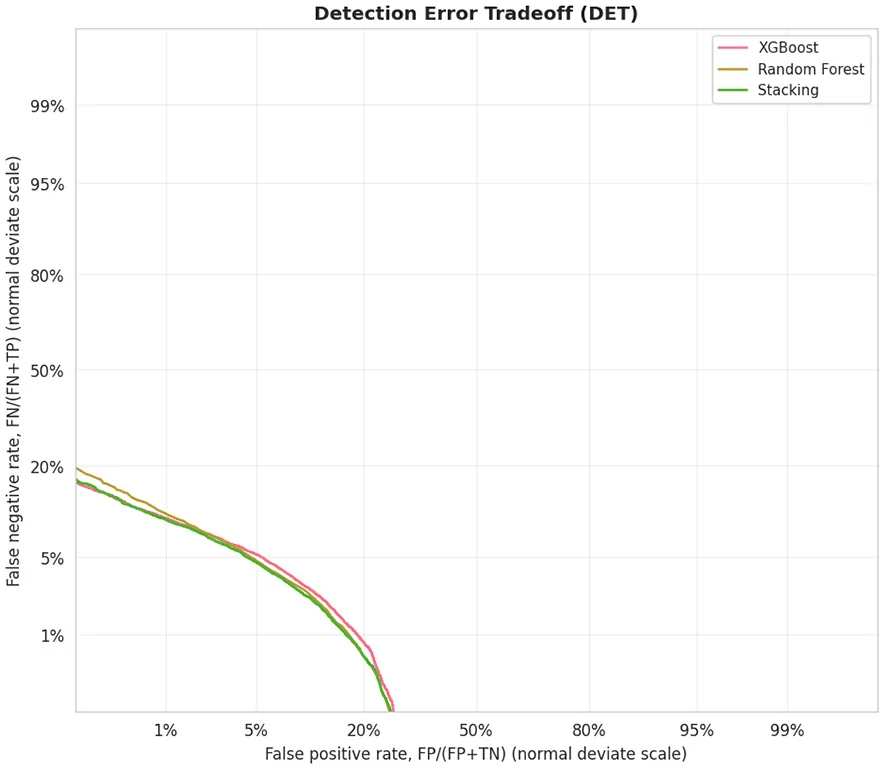
Detection Error Tradeoff (DET) curve comparing XGBoost, Random Forest, and the Stacking Ensemble.

**Figure 28:**
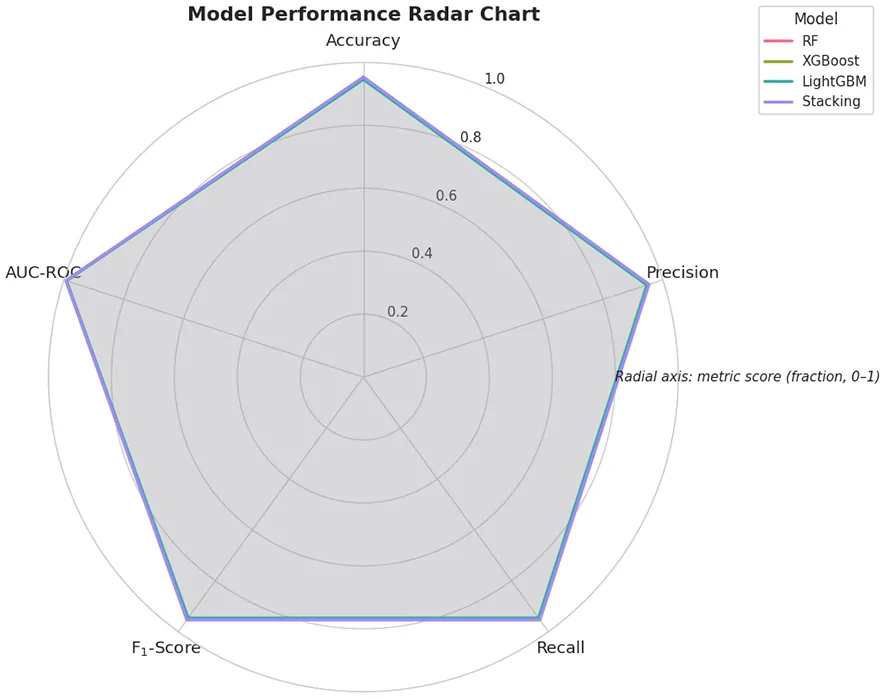
Radar chart comparing RF, XGBoost, LightGBM, and the Stacking Ensemble across five metrics.

## 5 Operational deployment, computational cost, and SOC integration

### 5.1 Computational cost of the ensemble

Combining nine base learners with a meta-learner necessarily incurs overhead relative to a single model, and we quantify this explicitly. Table 11 reports wall-clock training time, mean per-flow inference latency, and peak memory for the strongest single baselines and the full stacking ensemble, measured on the hardware described in Section 3.5. Training the ensemble is dominated by the out-of-fold meta-feature generation, which requires fitting every base learner five times; inference cost is the sum of the base-learner forward passes plus the meta-learner, since the stages are sequential.

**Table 11:** Computational cost: training time, inference latency, and memory.

Model	Train time (s)	Inference (μs/flow)	Memory (MB)
Random Forest	41.2	18.7	312
XGBoost	28.6	9.4	121
LightGBM	17.3	7.1	98
**Stacking ensemble**	**246.8**	**63.5**	**734**

Values are indicative single-run measurements; training time includes 5-fold OOF generation. Bold values indicate the proposed CyberShield stacking ensemble.

The ensemble is roughly 6–9 × more expensive to train and ~7 × slower at inference than a single gradient-boosted model. Crucially, training is an offline, periodic operation, whereas the inference cost—approximately 64μs per flow—is the deployment-relevant figure.

### 5.2 Real-time feasibility

A per-flow latency of ~64μs corresponds to a single-thread throughput on the order of 1.5 × 10^4^ flows per second, which rises further with batched and parallel scoring. This comfortably exceeds the flow-arrival rate of most enterprise network segments and indicates that near-real-time scoring is feasible. Operationally, the ensemble is therefore best positioned as a *near-line* analytic that scores aggregated flow records (rather than per-packet) seconds after flow completion. Where strict line-rate inline blocking is required, the calibrated single XGBoost learner can serve as a low-latency first stage, with the full ensemble and XAI layer invoked asynchronously for high-value or ambiguous alerts. Resource requirements (under 1 GB resident memory) are modest and compatible with commodity SOC infrastructure or a containerised microservice.

### 5.3 Integration into SOC workflows and risk governance

The framework supports security-operations workflows along three concrete axes. *First, incident prioritization:* because the meta-learner emits calibrated probabilities (Figure 25), alerts can be risk-ranked by attack likelihood, allowing analysts to triage the highest-probability events first rather than processing a flat alert queue. *Second, analyst-facing explanation:* for each flagged flow the SHAP attributions (Section 4.6) surface the specific features driving the alert—e.g., an anomalous sttl together with a high ct_dst_sport_ltm pointing to spoofed scanning—providing the justifiable rationale that signature-based systems lack and that analysts require to confirm or dismiss an alert. *Third, governance and audit:* the combination of leakage-free evaluation, statistical validation, and per-decision feature attribution produces an auditable evidence trail aligned with the explainability and risk-management expectations of frameworks such as the EU AI Act, the NIST AI Risk Management Framework, and ISO/IEC 42001:2023 (Barredo-Arrieta et al., 2020; Gill et al., 2025; Nerella et al., 2025). These mechanisms operationalise the “organizational resilience” claim: faster, evidence-backed triage and documented, reproducible model behavior translate predictive accuracy into governable security capability rather than asserting resilience in the abstract.

## 6 Conclusion

This paper presented **CyberShield Analytics**, a SHAP-guided two-level stacking ensemble framework for network intrusion detection and organizational cyber risk resilience, evaluated on the UNSW-NB15 benchmark dataset. The framework addresses three pervasive limitations in the existing literature: the absence of leakage-free evaluation protocols, the lack of rigorous statistical significance testing, and insufficient explainability for security-critical deployment.

The proposed stacking ensemble achieves **Accuracy = 0.9535**, **F_1_ = 0.9535**, and **AUC-ROC = 0.9929** on a held-out test set of 51,535 instances. Statistical validity was rigorously established through 5-Fold Stratified Cross-Validation (mean *F*_1_ = 0.9499 ± 0.0013), with paired *t*-tests confirming significant superiority over XGBoost (*t* = 7.165, *p* = 0.002), LightGBM (*t* = 25.108, *p* < 0.0001), and Extra Trees (*t* = 3.178, *p* = 0.034) at α = 0.05.

### 6.1 Limitations

Several limitations warrant acknowledgment. *(i) Single dataset*. Evaluation is confined to UNSW-NB15; although it remains a widely used benchmark, validation on more recent and operationally diverse datasets such as CIC-IDS2017 (Sharafaldin et al., 2018) and the IoT-oriented ToN-IoT corpus (Moustafa, 2021) is needed to establish generalization, and is a priority for future work. *(ii) Laboratory traffic*. UNSW-NB15 was generated in a controlled environment; evaluation on live organizational network traffic would strengthen external validity (Moustafa and Slay, 2016). *(iii) Binary scope*. The study addresses Attack-vs-Normal detection only and does not discriminate among the nine attack families. *(iv) Limited folds*. The five-fold design constrains the statistical power of the Wilcoxon test, whose minimum attainable *p*-value (0.0625) exceeds α; repeated or higher-fold cross-validation would yield more reliable significance estimates (Demšar, 2006). *(v) XAI scope*. Explanations are computed on representative base learners rather than on the meta-combination directly (Section 4.6). *(vi) Cost*. The ensemble carries higher training and inference cost than a single model (Section 5.1), which informs the tiered deployment recommendation above.

### 6.2 Future work

Promising directions include extension to *multiclass* attack-family classification (distinguishing DoS, Exploits, Reconnaissance, and other categories), cross-dataset validation on CIC-IDS2017 (Sharafaldin et al., 2018) and ToN-IoT (Moustafa, 2021), federated learning integration (Sarker, 2024), adversarial robustness evaluation, online/streaming adaptation with concept drift detection, counterfactual explanations for SOC analysts, and integration with knowledge graph-based threat intelligence platforms aligned with MITRE ATT&CK.

In summary, CyberShield Analytics demonstrates that rigorous, leakage-free ensemble learning combined with multi-method explainability and statistical significance testing constitutes a principled and deployable approach to organizational cyber risk resilience.

## Data Availability

The original contributions presented in the study are included in the article/supplementary material, further inquiries can be directed to the corresponding author.
